# Deciphering a Marine Bone-Degrading Microbiome Reveals a Complex Community Effort

**DOI:** 10.1128/mSystems.01218-20

**Published:** 2021-02-09

**Authors:** Erik Borchert, Antonio García-Moyano, Sergio Sanchez-Carrillo, Thomas G. Dahlgren, Beate M. Slaby, Gro Elin Kjæreng Bjerga, Manuel Ferrer, Sören Franzenburg, Ute Hentschel

**Affiliations:** a GEOMAR Helmholtz Centre for Ocean Research Kiel, RD3 Research Unit Marine Symbioses, Kiel, Germany; b NORCE Norwegian Research Centre, Bergen, Norway; c CSIC, Institute of Catalysis, Madrid, Spain; d Department of Marine Sciences, University of Gothenburg, Gothenburg, Sweden; e IKMB, Institute of Clinical Molecular Biology, University of Kiel, Kiel, Germany; f Christian-Albrechts University of Kiel, Kiel, Germany; MIT

**Keywords:** *Osedax mucofloris*, bone biome, bone degradation, metagenomics

## Abstract

The marine bone biome is a complex assemblage of macro- and microorganisms; however, the enzymatic repertoire to access bone-derived nutrients remains unknown. The bone matrix is a composite material made up mainly of organic collagen and inorganic hydroxyapatite. We conducted field experiments to study microbial assemblages that can use organic bone components as nutrient source. Bovine and turkey bones were deposited at 69 m depth in a Norwegian fjord (Byfjorden, Bergen). Metagenomic sequence analysis was used to assess the functional potential of microbial assemblages from bone surface and the bone-eating worm Osedax mucofloris, which is a frequent colonizer of whale falls and known to degrade bone. The bone microbiome displayed a surprising taxonomic diversity revealed by the examination of 59 high-quality metagenome-assembled genomes from at least 23 bacterial families. Over 700 genes encoding enzymes from 12 relevant enzymatic families pertaining to collagenases, peptidases, and glycosidases putatively involved in bone degradation were identified. Metagenome-assembled genomes (MAGs) of the class *Bacteroidia* contained the most diverse gene repertoires. We postulate that demineralization of inorganic bone components is achieved by a timely succession of a closed sulfur biogeochemical cycle between sulfur-oxidizing and sulfur-reducing bacteria, causing a drop in pH and subsequent enzymatic processing of organic components in the bone surface communities. An unusually large and novel collagen utilization gene cluster was retrieved from one genome belonging to the gammaproteobacterial genus *Colwellia*.

**IMPORTANCE** Bones are an underexploited, yet potentially profitable feedstock for biotechnological advances and value chains, due to the sheer amounts of residues produced by the modern meat and poultry processing industry. In this metagenomic study, we decipher the microbial pathways and enzymes that we postulate to be involved in bone degradation in the marine environment. We here demonstrate the interplay between different bacterial community members, each supplying different enzymatic functions with the potential to cover an array of reactions relating to the degradation of bone matrix components. We identify and describe a novel gene cluster for collagen utilization, which is a key function in this unique environment. We propose that the interplay between the different microbial taxa is necessary to achieve the complex task of bone degradation in the marine environment.

## Abstract (precis)

Bones are an underexploited, yet potentially profitable feedstock for biotechnological advances and value chains, due to the sheer amounts of residues produced by the modern meat and poultry processing industry. In this metagenomic study, we decipher the microbial pathways and enzymes that we postulate to be involved in bone degradation in the marine environment.

## INTRODUCTION

The marine environment is a treasure trove for novel microbial assemblages and organic catalysts (enzymes) (1–3). The oceans cover approximately 70% of the Earth’s surface with an estimated volume of about 2 × 10^18^ m^3^, and due to their incredible environmental variability (e.g., temperature, pressure, salinity, light availability), they have sparked the evolution of an unprecedented range of different microbes and hence enzymatic activities (4–6). Genome sequencing of individual microbial isolates of complex communities has allowed us to get a glimpse of their diversity and their potential functions in multiple environmental contexts. The lack of cultivable microbes has further driven the development of functional and sequence-driven metagenomic analyses and enabled us to decipher complex interactions in entire microbial consortia (7–9).

The deep sea was for a long time seen as an almost lifeless environment, as no one could imagine life to be possible under conditions vastly different from shallower ocean waters in respect to nutrient and energy resources. Nowadays, we know that even the deep sea is teeming with life; hydrothermal vents, sponge grounds, and coral gardens are recognized as examples of unique and complex habitats (10–12). Nonetheless, the deep sea is also a harsh environment with limited nutrient sources. In this respect, sudden events like a whale fall create a locally defined but significant nutrition source for deep-sea life that can last for years or even decades (13). These whale carcasses are rapidly stripped of their soft tissue by scavengers (i.e., hagfish, sleeper sharks, rat tail fish, and crabs), but the energy-rich bones and cartilage remain as a recalcitrant nutrient source. More than 15 years ago, *Osedax* was described, a genus of bone-eating annelid worms (14), and has since then been investigated for its diversity and ecology and how it accesses the organic compounds of whale bones (14–17). These worms are gutless and rather bore cavities into bones and develop a root tissue in these cavities for food intake. Furthermore, this evolutionarily novel and specialized organ was shown to harbor endosymbionts, typically affiliated with Oceanospirillales (14, 18–21). *Osedax* species are known for their ability to acidify their environment via elevated expression levels of vacuolar H^+^-ATPase (VHA) specifically in their root tissue and of carbonic anhydrase (CA) throughout their body, to dissolve hydroxyapatite and access collagen and lipids from the bone matrix (16). Miyamoto et al. found a high number of matrix metalloproteinases in the genome of Osedax japonicus compared to other invertebrates, potentially assisting in digestion of collagen and other proteins derived from bones (15). The species can thus be regarded as a member of the bone biome and an important facilitator in this degradation process. In the northern North Atlantic, Osedax mucofloris was described in 2005 and has been shown to consistently colonize bone material on the sea floor below a depth of 30 m (22, 23).

Bone is a recalcitrant and heterogeneous composite material made of a mineral phase, an organic phase, and water. Hydroxyapatite crystals in the mineral phase contribute to the structural strength in bones. The organic phase includes proteins, such as collagen and structural glycoproteins (e.g., proteins decorated with sugars such as mannose, galactose, glucosamine, galactosamine, *N*-acetylglucosamine, *N*-acetylgalactosamine, rhamnose, sialic acid, and fucose), lipids, and cholesterol composed of various triglycerides (24–26). Up to 90% of the protein content in mature bone is made of type I collagen, a triple-helical molecule rich in glycine, hydroxyproline, and proline that assembles into fibrils with a high number of hydrogen bonds, hydrophobic interactions, and covalent cross-linking, which together confer high structural stability to collagen fibrils (27). Due to this structural and chemical complexity, it is expected that the degradation of the recalcitrant bone matrix will require a synergistic multienzyme system and require a microbial community effort. Similar multienzyme systems are well described, for example, for the degradation of lignocellulose, another important organic polymer (28, 29). This system will likely include essential enzymes in the breakdown of the organic matrix, namely, collagenases that break the peptide bonds in collagen and other proteases/peptidases (endo- and exopeptidases) that attack the glycoproteins. Furthermore, neuraminidases (sialidases), α-mannosidases, α/β-galactosidases, α-fucosidase, α-rhamnosidase, and α/β-*N*-acetylhexosaminidase (glucose and galactose-like), all glycoside hydrolase enzymes, are likely involved in cleavage of glycosidic linkages. Finally, in the digestion of the cholesterol-containing marrow, cholesterol oxidases may be involved.

To date, only a few studies have been published that focus on microbial communities to understand the necessary complex interactions in bone degradation, mainly relying on 16S rRNA gene sequencing data (30–33) and one metagenomic study of a whale fall (34). We here provide a first comprehensive overview and identify putative key functions involved in bone degradation of the marine bone microbiome retrieved from deployed bone material, including microbial communities from the gutless worm *Osedax mucofloris* and free-living microbial assemblages developing on the bone surface.

## RESULTS

### Recovery of artificially deployed bone for bone microbiome metagenomic analysis.

Turkey and bovine bones were deployed at 69 m depths in Byfjorden, a fjord outside Bergen, Norway. After 9 months of incubation, underwater images taken by a remotely operated vehicle (ROV) showed microbial colonization of the bone surfaces (see Fig. S1A in the supplemental material). *Osedax mucofloris* worms were observed, especially on the joints, and in some cases forming small colonies of several individuals inside a single cavity under the external bone tissue. Although not the subject of this study, a larger diversity of invertebrate fauna including *Ophryotrocha*, *Vigtorniella*, and *Capitella* worms were also observed. Dense microbial mats developed asymmetrically with preference for the joint adjacent sections (epiphysis), which also appeared in aquarium settings (Fig. S1B).

10.1128/mSystems.01218-20.1 FIG S1 (A) ROV image of bone incubation experiment in the Byfjorden at 68-m depth; shown is a cow tibia. (B) Bones retrieved from the Byfjorden after 9 months of incubation; bacterial mats and blackening at the epiphysis can be seen. Download FIG S1, TIF file, 1.1 MB. Copyright © 2021 Borchert et al. 2021 Borchert et al. This content is distributed under the terms of the Creative Commons Attribution 4.0 International license.

Two sets of samples, an *Osedax-*associated bone microbiome (OB) and a bone surface-associated biofilm (BB), were collected, each consisting of four individual metagenomes (Table 1). The coassemblies of each sample set comprised >300,000 contigs and 342.7 Mb for the OB-metagenomes and >1,000,000 contigs and 1.22 Gb for the BB-metagenomes, respectively, considering only contigs >500 bp (Table S1). The individual metagenomes were profiled separately with Kaiju (35), a database-aided metagenomic read taxonomy classifier. The OB-metagenomes comprised 45 to 76% bacterial, 15 to 34% eukaryotic, and 0.4 to 0.5% archaeal reads, and the BB-metagenomes are made up of 92 to 95% bacterial, 2 to 4% eukaryotic, and 0.4 to 0.7% archaeal reads (Table S1).

**TABLE 1:** Metagenome sampling information and number of retrieved metagenome-assembled genomes (MAGs)

Sample	Sample type	Bone type, organism	Collection dates^*a*^	Sampling location (GPS)	No. of high-quality MAGs
A5	*Osedax mucofloris*	Femur, turkey (Meleagris gallopavo)	08.01.2017	Byfjorden, Bergen, Norway (60.397093N; 5.301293E)	15 (OB)
A9			08.02.2017		
A9n			08.02.2017		
B4			14.04.2017		
D1	Bone surface biofilm communities	Tibia, cow (Bos taurus)	02.2017		44 (BB)
D2			11.12.2017		
I1			27.01.2017		
I3			11.12.2017		

a Shown as day.month.year, or month.year for sample D1.

10.1128/mSystems.01218-20.5 TABLE S1 Metagenome statistics. Download Table S1, DOCX file, 0.01 MB. Copyright © 2021 Borchert et al. 2021 Borchert et al. This content is distributed under the terms of the Creative Commons Attribution 4.0 International license.

### Metagenome-assembled genomes (MAGs) from the marine bone microbiome display taxonomic diversity and novelty.

**FIG 1:**
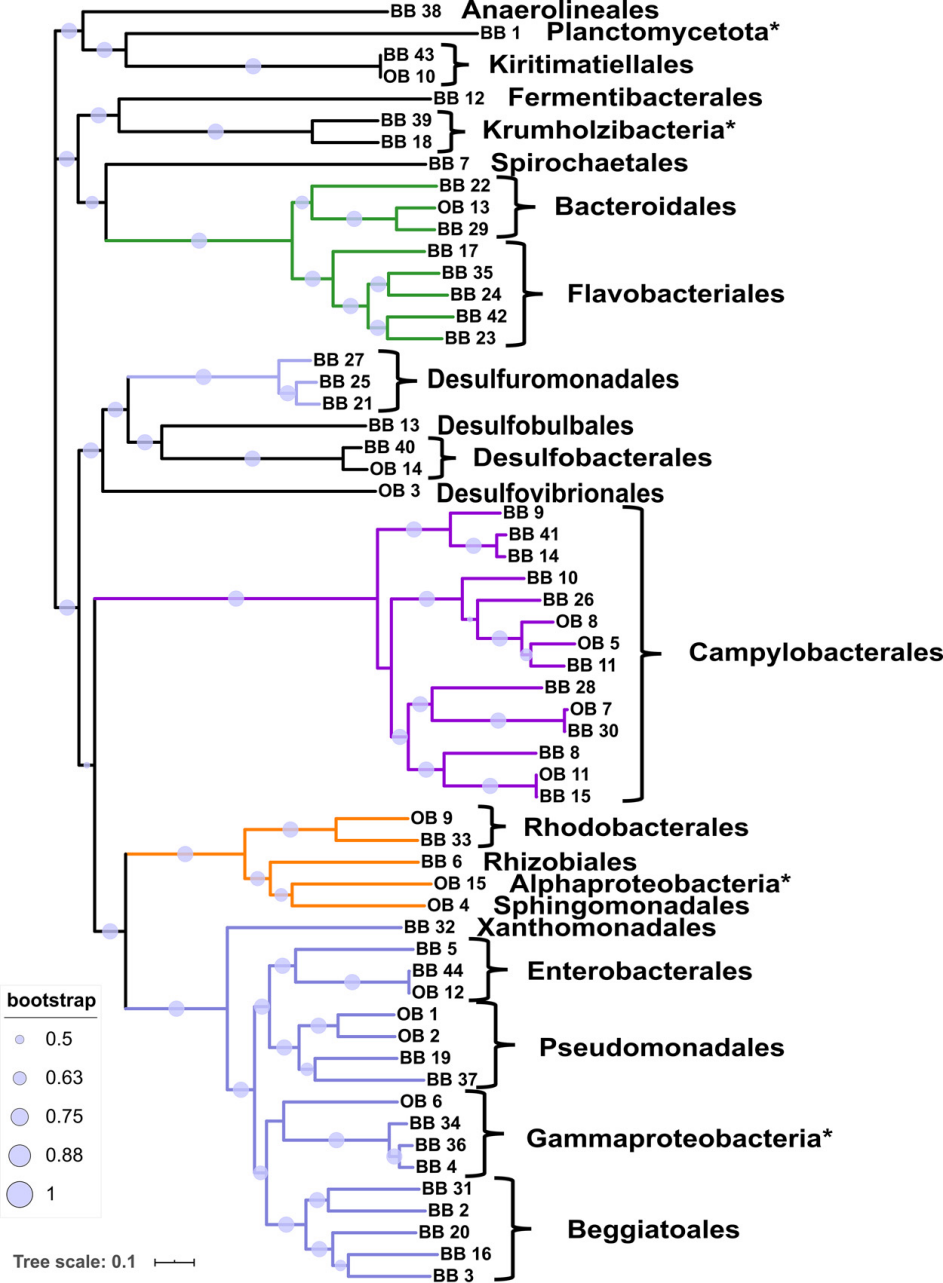
Phylogenomic maximum likelihood tree of the obtained 59 high-quality MAGs from *Osedax*-associated bone microbiome (OB) and bone surface-associated biofilm (BB). Bootstrap values greater than 0.5 are displayed as circles on the branches. The five most common bacterial classes are colored (green, Bacteroidia; light purple, Desulfuromonadia; purple, Campylobacteria; orange, Alphaproteobacteria; and blue, Gammaproteobacteria). Order-level identifications are listed, and MAGs for which only class-level identification could be inferred are marked with an asterisk.

Fifty-nine high-quality MAGs (Fig. 1) (>90% completion and <10% redundancy) were extracted from the coassembled metagenomes (see Table S3 for MAG sequence statistics). The MAGs span 11 phyla, 14 classes, 19 orders, and at least 23 families. About 63% of the MAGs (37/59) possess genomic novelty as determined by their relative evolutionary divergence (RED) (36) to their closest common ancestor (Table S2). One MAG could be identified only up to phylum level, seven to class level, seven to order level, 18 up to family level, and four up to genus level. The taxonomy of most MAGs was fully resolved based on 120 marker genes. The three best represented phyla were Proteobacteria (22 MAGs), Campylobacterota (14 MAGs), and Bacteroidota (8 MAGs). However, the percental distribution of the most abundant classes differs between the two metagenome sets. The OB-MAGs were dominated by the classes Gammaproteobacteria (27%), Campylobacteria (27%), and Alphaproteobacteria (20%), while the BB-MAGs were mainly affiliated with Gammaproteobacteria (30%), Campylobacteria (23%), and Bacteroidia (16%).

10.1128/mSystems.01218-20.6 TABLE S2 Taxonomic affiliation of MAGs according to the work of Parks et al. (2018) and taxonomic novelty identified by RED (*) (36). Download Table S2, DOCX file, 0.02 MB. Copyright © 2021 Borchert et al. 2021 Borchert et al. This content is distributed under the terms of the Creative Commons Attribution 4.0 International license.

10.1128/mSystems.01218-20.7 TABLE S3 MAG sequence data. All MAGs labeled “OB” were retrieved from *Osedax* samples, and all MAGs labeled with “BB” were from bone surface biofilms. Download Table S3, DOCX file, 0.02 MB. Copyright © 2021 Borchert et al. 2021 Borchert et al. This content is distributed under the terms of the Creative Commons Attribution 4.0 International license.

### Sulfur cycling in the marine bone microbiome.

**FIG 2:**
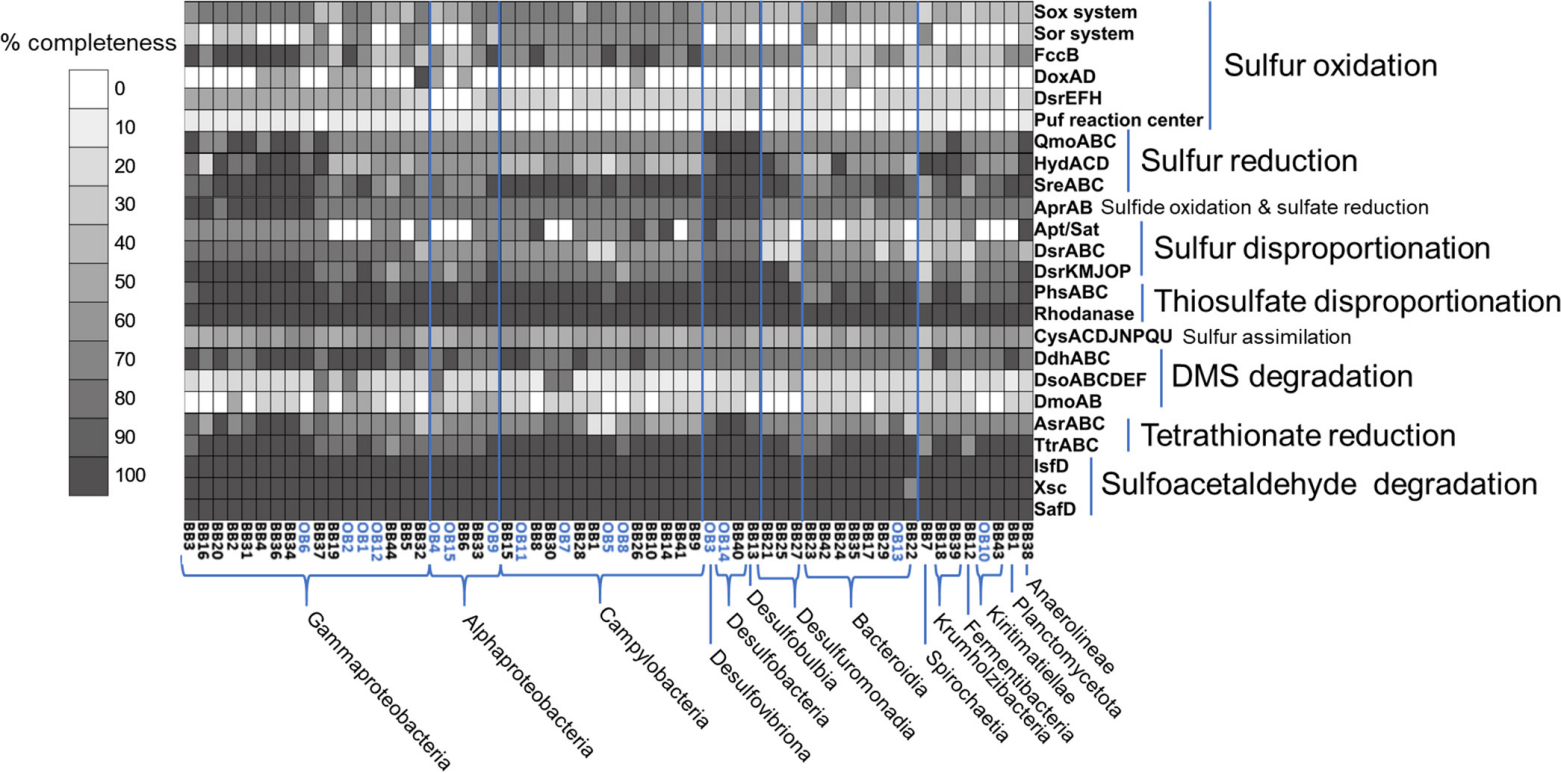
Whole-genome metabolic pathway comparison for genes of the sulfur metabolism. Analysis was done with MEBS (37), and MAGs were phylogenetically grouped according to the GTDB-Tk pipeline (36). The gray scale represents the completeness of a given pathway or multienzyme system shown in the heatmap for each MAG. The OB MAGs are highlighted in blue. The “Sox system” is constituted by the *sox*XYZABCD genes, and the “Sor system” consists of *sor*ABDE.

All MAGs were investigated using the multigenomic entropy-based score pipeline (MEBS) (37) for their ability to utilize sulfur as energy source via the abundance of selected marker genes for related pathways (Fig. 2). Sulfur cycling is of relevance to bone degradation due to the generation of free protons by sulfur and sulfur compound oxidation processes (thiotrophy), which leads to an acidification.

MAGs affiliated with the order Campylobacterales encode almost complete Sox (*sox*XYZABCD) and Sor (*sor*ABDE) enzyme systems for sulfur oxidation. Furthermore, Gammaproteobacteria affiliated with Beggiatoaceae and other unclassified Gammaproteobacteria are all potentially capable of thiotrophy via utilization of reduced sulfur compounds as electron donors (flavocytochrome c sulfide dehydrogenase [(*fcc*B] and adenosine-5′-phospho-sulfate reductase [*apr*AB]) and partial predicted Sox sulfur/thiosulfate oxidation pathway. MAGs identified as Desulfuromonadia, Desulfobacteria, Desulfobulbia, and Desulfovibrionia possess marker genes for sulfur reduction (*qmo*ABC, *hyd*ACD, *sre*ABC) and lack Sox and Sor pathway genes, all of which belong to the new proposed phylum of Deltaproteobacteria (38). In all Gammaproteobacteria (except BB32), Desulfobacteria, Desulfobulbia, and Desulfovibrionia, genes for dissimilatory sulfite reductase (*dsr*ABC) are present. Müller et al. (2015) described that gammaproteobacterial *dsr*AB-type genes are commonly involved in oxidative reactions, whereas *dsr*AB in Desulfobacterota are reductive-type *dsr*AB (39). All MAGs contain at least partial pathways for dissimilatory tetrathionate reduction (*ttr*ABC), thiosulfate disproportionation (*phs*ABC and rhodanase), and dimethylsulfide (DMS) degradation (*ddh*ABC) and contain also genes for sulfoacetaldehyde degradation (*isf*D, *xsc*, and *saf*D). In addition, the phenotypic trait of H_2_S production was identified in 10 MAGs (Traitar analysis [40]), two of which were Marinifilaceae, two Krumholzibacteria, two *Sulfurospirillum*, one Spirochaetaceae, two Desulfobacteraceae, and one *Pseudodesulfovibrio*.

**FIG 3:**
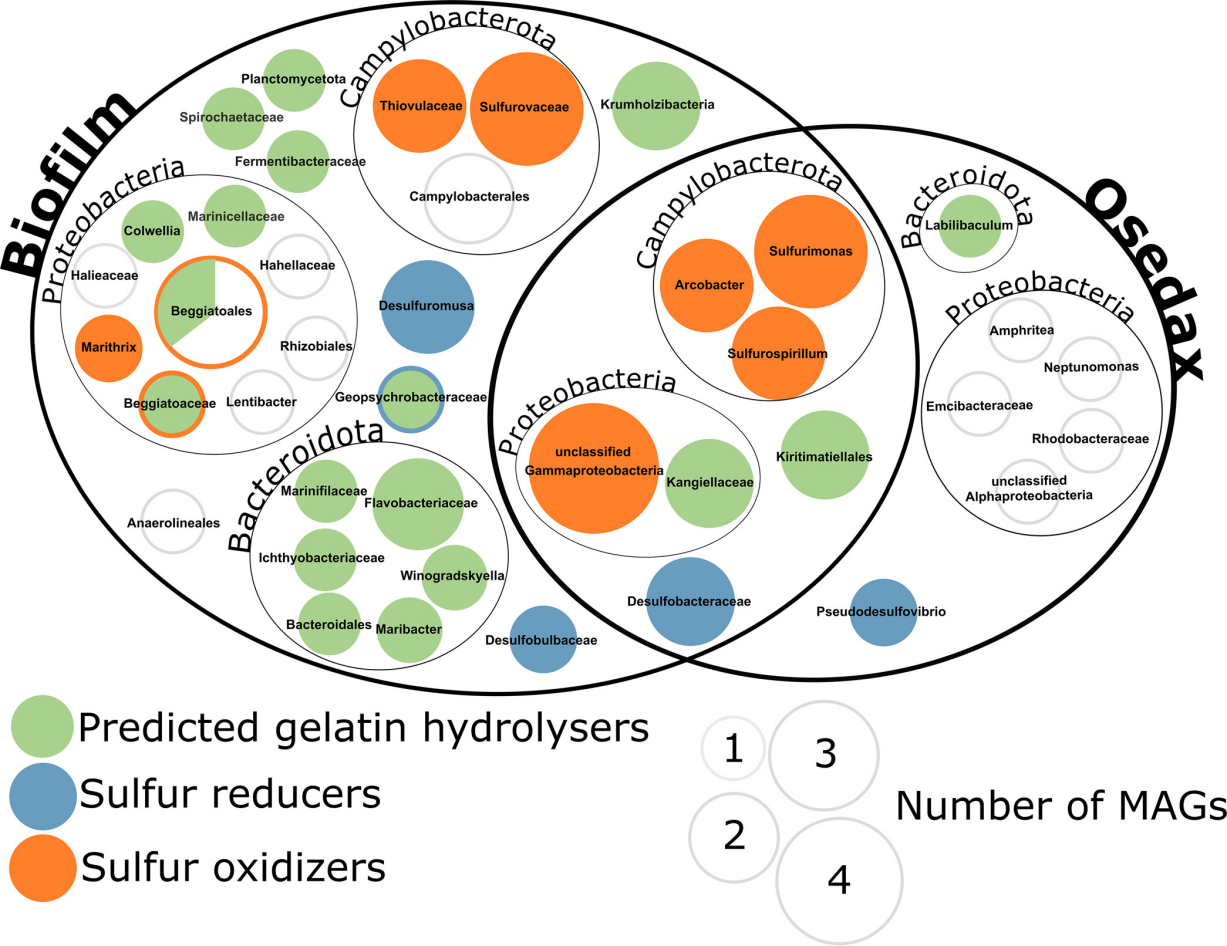
Abundance and taxonomic affiliation of predicted gelatin hydrolyzers (green) (Traitar) in *Osedax* and biofilm-derived MAGs. Additionally, sulfur reducers (blue) and sulfur oxidizers (orange) (MEBS) are shown, while white indicates absence of these traits. MAGs are displayed at the deepest taxonomic classification obtained. The size of the circles reflects the number of MAGs within each clade.

The anticipated thiotrophy has the potential to contribute massively to the acidification of the environment via the oxidation of reduced sulfur compounds leading to production of sulfuric acid (41). This requires a close interaction between sulfur-reducing bacteria (SRB) producing hydrogen sulfide and sulfur-oxidizing bacteria (SOB) utilizing the hydrogen sulfide, while releasing protons (Fig. 3). The Traitar analysis identified 10 MAGs potentially able to produce hydrogen sulfide, including known SRB like Desulfobacteraceae, *Pseudodesulfovibrio*, and others like *Sulfurospirillum* (42–44). The bone microbiome is especially enriched in taxa containing known SOB, like the large filamentous bacteria Beggiatoales (5 MAGs) (45) and Campylobacterales (10 MAGs) (46). Furthermore, one MAG identified as Desulfobulbaceae was found in the bone-associated metagenomes. Members of this group are known to be able to perform sulfur oxidation and sulfur reduction (47, 48).

### Acidification by carbonic anhydrases.

Carbonic anhydrases were identified in 51 of 59 MAGs. Nineteen out of 94 carbonic anhydrases contained a signal peptide for extracellular export (16 MAGs). Fifteen were predicted to contain a Sec signal peptide (SPI) and four to encode lipoprotein (SPII) signal peptides. Four out of five Beggiatoales MAGs were predicted to contain carbonic anhydrases with an SPI signal peptide (BB2, BB3, and BB20) or an SPII signal peptide (BB16). The remaining three SPII signal peptides were found in carbonic anhydrases from Campylobacterales (BB8, BB10, and BB11). Interestingly, BB8 contains at least three carbonic anhydrases, one with an SPI, one with an SPII, and one where a signal peptide was not predicted. Three SPI signal peptides were found in carbonic anhydrases from unclassified gammaproteobacteria (BB4, BB34, and BB36). The remaining SPI including carbonic anhydrases were found in five Campylobacterales MAGs (BB14, BB26 [three carbonic anhydrases and two containing SPI signal peptides], BB30, BB41, and OB7) and one Desulfobulbaceae MAG (BB13). Based on phylogenetic relationship to known carbonic anhydrases described in the work of Capasso and Supuran (49), 18 out of 19 carbonic anhydrases belong to the α-carbonic anhydrase family and one to the β-family, with no γ-family carbonic anhydrases found (Fig. S2).

10.1128/mSystems.01218-20.2 FIG S2 Maximum-likelihood tree of all 94 obtained carbonic anhydrases and relevant reference sequences from the work of Capasso and Supuran, 2015 (49). Download FIG S2, TIF file, 2.8 MB. Copyright © 2021 Borchert et al. 2021 Borchert et al. This content is distributed under the terms of the Creative Commons Attribution 4.0 International license.

10.1128/mSystems.01218-20.3 FIG S3 Genomic context of all identified M9 collagenase in the investigated MAGs. M9 collagenases are encircled in red, and all genes potentially involved in collagen/proline utilization pathways are encircled in green. The graphic was made with SnapGene software (from GSL Biotech; information available at snapgene.com). Download FIG S3, TIF file, 2.4 MB. Copyright © 2021 Borchert et al. 2021 Borchert et al. This content is distributed under the terms of the Creative Commons Attribution 4.0 International license.

10.1128/mSystems.01218-20.4 FIG S4 Comparison of the bone microbiome to seawater metagenomes. Tara Oceans seawater MAGs have been chosen for comparison. The Tara Oceans data set comprises here 832 bacterial MAGs with >70% completion. The bone microbiome data set has been rebinned to >70% completion and contains now 86 MAGs for this comparison. (A) Percental bacterial class abundance comparison between bone microbiome MAGs (orange) and Tara Oceans MAGs (blue). Only bacterial classes present within the bone microbiome have been included in this graph; all classes present only in the Tara Oceans data set are combined in “Others.” (B) Enzyme abundance comparison. Shown is the ratio per MAG for each investigated enzyme class. All MAGs have been profiled with the here-established HMM profiles, and the total obtained number of enzymes was divided by the number of MAGs per data set. Highlighted in red are the higher ratios and in bold red ratios at least twice as high as in the other data set. Download FIG S4, TIF file, 2.5 MB. Copyright © 2021 Borchert et al. 2021 Borchert et al. This content is distributed under the terms of the Creative Commons Attribution 4.0 International license.

### Gelatin hydrolysis.

With respect to microbial bone degradation, the phenotypic feature of gelatin hydrolysis was analyzed using the Traitar software (40), which provides genome-informed phenotype predictions. Twenty-two MAGs showed capacity for gelatin hydrolysis (19 MAGs in the bone surface community [BB] and three in the *Osedax*-associated communities [OB]). With gelatin being a primarily bone collagen-derived compound, we consider gelatin hydrolysis a key trait for the microbial community studied here. All eight Bacteroidia-affiliated MAGs possess the gelatin hydrolysis trait, as do seven Gammaproteobacteria MAGs, one tentative Planctomycetota MAG, one Spirochaetia MAG, two Krumholzibacteria MAGs, one Thiovulaceae MAG, one Geopsychrobacteraceae MAG, and one Fermentibacteria MAG (Fig. 3).

### Enzymes involved in bone degradation.

**FIG 4:**
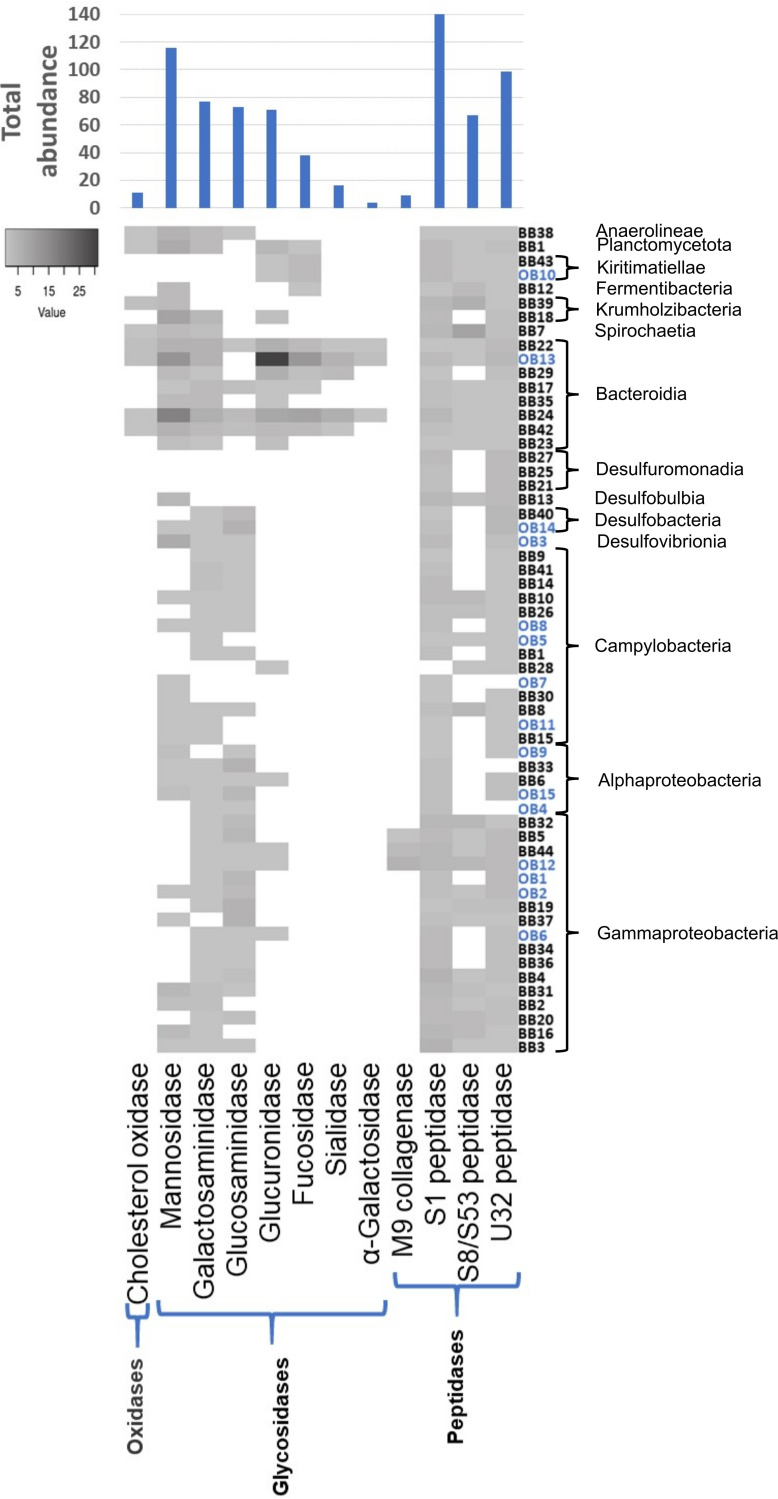
Abundance heatmap of the 12 investigated enzyme COG classes in the 59 bone degradome MAGs. The MAGs are arranged according to their taxonomic affiliation. The absolute abundance of each enzyme COG class is depicted in the diagram on top of the heatmap. The OB MAGs are highlighted in blue.

Based on the structure and composition of mature vertebrate bone tissue, we hypothesized that 12 different Clusters of Orthologous Groups (COGs) and peptidase/collagenase families were relevant for the enzymatic attack of the bone organic matrix. This “bone-degradome” comprised the peptidase families S1 (COG0265), S8/S53 (and Pfam00082), U32 (COG0826), and M9 collagenase (including Pfam01752), mannosidases (COG0383), sialidases (COG4409), glucuronidases (COG3250), glucosaminidases (COG1472), galactosaminidases (COG0673), α-galactosidases (Pfam16499), cholesterol oxidases (COG2303), and fucosidases (COG3669) (choice of enzymes is justified in Materials and Methods). We constructed hidden Markov model (HMM) profiles that were used to screen the abundance of each enzyme family in all MAGs (Fig. 4). In total 722 enzymes belonging to the 12 investigated enzyme families were identified in the 59 MAGs. The glycosidase families of mannosidases, galactosaminidases, and glucosaminidases and the peptidase families S1, S8/S53, and U32 were widespread in the MAGs (Fig. 4). M9 collagenases and α-galactosidases (Pfam16499) were found in only three MAGs. The M9 collagenases were solely found in Enterobacterales (BB5, BB44, and OB12). Pfam16499 was identified only in Bacteroidales (BB22, BB24, and OB13). The most abundant group of enzymes were the S1 peptidases (141 hits), followed by galactosaminidases (COG0673) (116 hits) and U32 peptidases (99 hits) (Fig. 4), constituting 20%, 16%, and 14% of all identified bone degrading enzymes, respectively. In general, Bacteroidales (BB17, BB22, BB24, BB29, BB42, and OB13) displayed the most diverse set of enzyme families related to bone degradation, as they contained genomic evidence of all enzymes besides M9 collagenases. MAGs belonging to the orders Desulfuromonadia, Desulfobulbia, Desulfobacteria, Desulfovibrio, and Campylobacteria (all of them driving the sulfur biogeochemical cycle), as well as some undefined alphaproteobacteria and gammaproteobacteria, appear to have no or few mannosidases (COG0383), glucuronidases (COG3250), fucosidases (COG3669), sialidases (COG4409), and α-galactosidases (Pfam16499).

### Collagen degradation.

**FIG 5:**
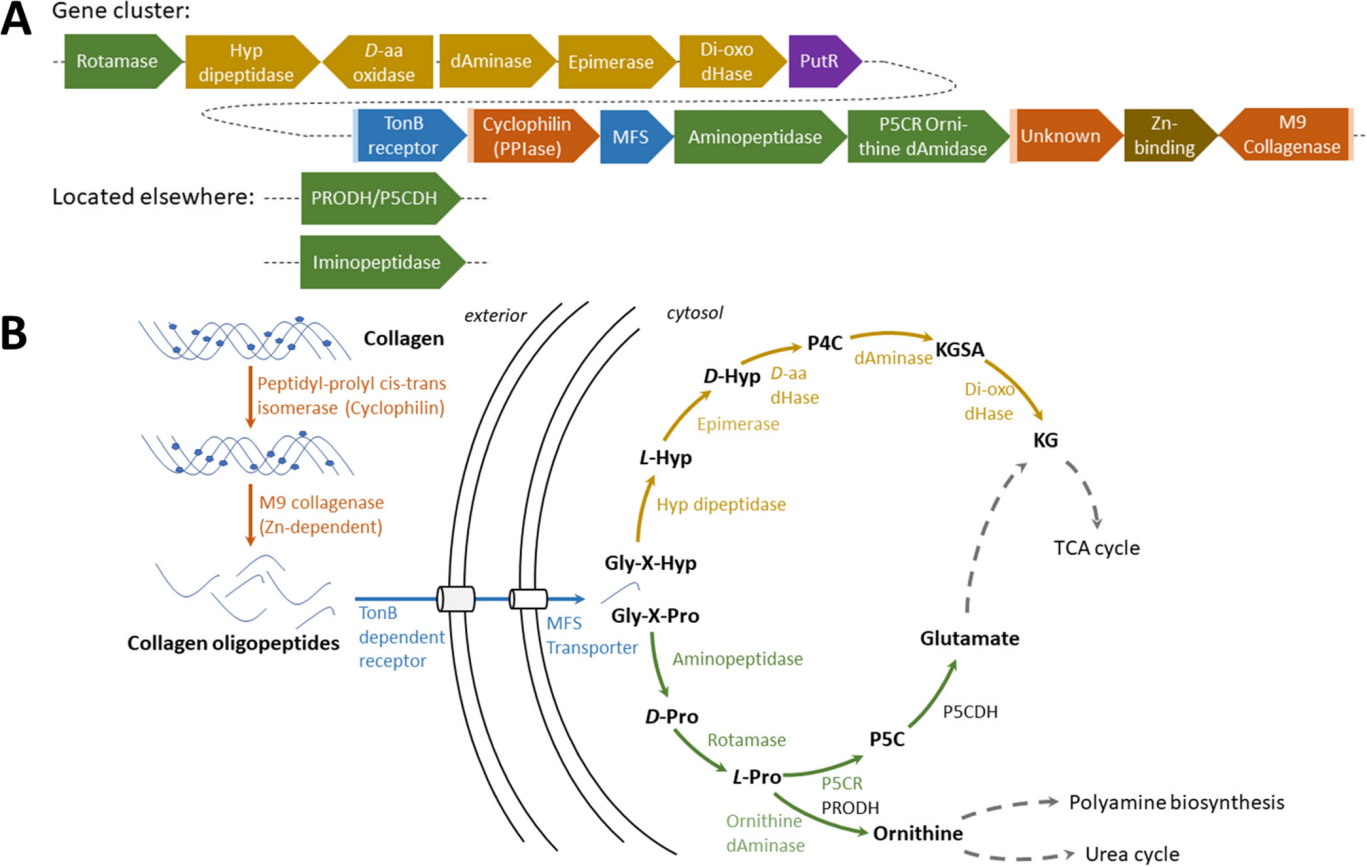
Collagen utilization pathway scheme in MAG BB5. (A) The gene cluster in BB5 spans approximately 21 kb, comprising 15 genes for collagen utilization, each color-coded respective to its functional group: orange for collagen hydrolysis, blue for uptake and transport, green for proline (Pro) utilization, ocher for hydroxyproline (Hyp) utilization, and brown for unknown function. The purple box is indicative of a signal peptide for secretion. (B) Metabolic model for collagen utilization in *Colwellia* BB5. Arrows and genes are color-coded in the same functional groups as in panel A. Dashed arrows point to a major metabolic pathway. Metabolite abbreviations: P4C (1-pyrroline 4-hydroxy-2-carboxylate), KGSA (alpha-ketoglutarate semialdehyde), KG (alpha-ketoglutarate), P5C (1-pyrroline-5-carboxylate). Enzyme abbreviations: d-aa dHase (d-hydroxyproline dehydrogenase), dAminase (pyrroline-4-hydroxy-2-carboxylate deaminase), di-oxo dHase (KGSA dehydrogenase), P5CR/ornithine dAminase (bifunctional 1-pyrroline-5-carboxylate reductase/ornithine cyclodeaminase), PRODH (proline dehydrogenase), P5CDH (pyrroline-5-carboxylate dehydrogenase).

We investigated the genomic context of each M9 collagenase for potential links to metabolic pathways, such as proline utilization (Fig. S3). *Colwellia* MAG BB5 possessed an approximately 21-kbp-long gene cluster presumably devoted to collagen utilization, which is unique in the data set and in the public databases. The functional cluster spans at least 15 different genes (Fig. 5A), featuring a secreted Zn-dependent M9 collagenase, a secreted peptidyl-prolyl *cis-trans* isomerase (cyclophilin-type PPIase [peptidyl-prolyl *cis-trans* isomerase]), a secreted unknown protein, and an unknown Zn/Fe chelating domain-containing protein. Additionally, one putative transporter (major facilitator superfamily [MFS] family), a TonB-dependent receptor, and several genes involved in the catabolism of proline and hydroxyproline, e.g., prolyl-aminopeptidase YpdF, intracellular peptidyl-prolyl *cis-trans* isomerase (rotamase), pyrroline reductase, hydroxyproline dipeptidase, 4-hydroxyproline epimerase, and others. Moreover, genes involved in transcription regulation such as PutR and the stringent starvation proteins A and B were identified in the same putative gene cluster.

**FIG 6:**
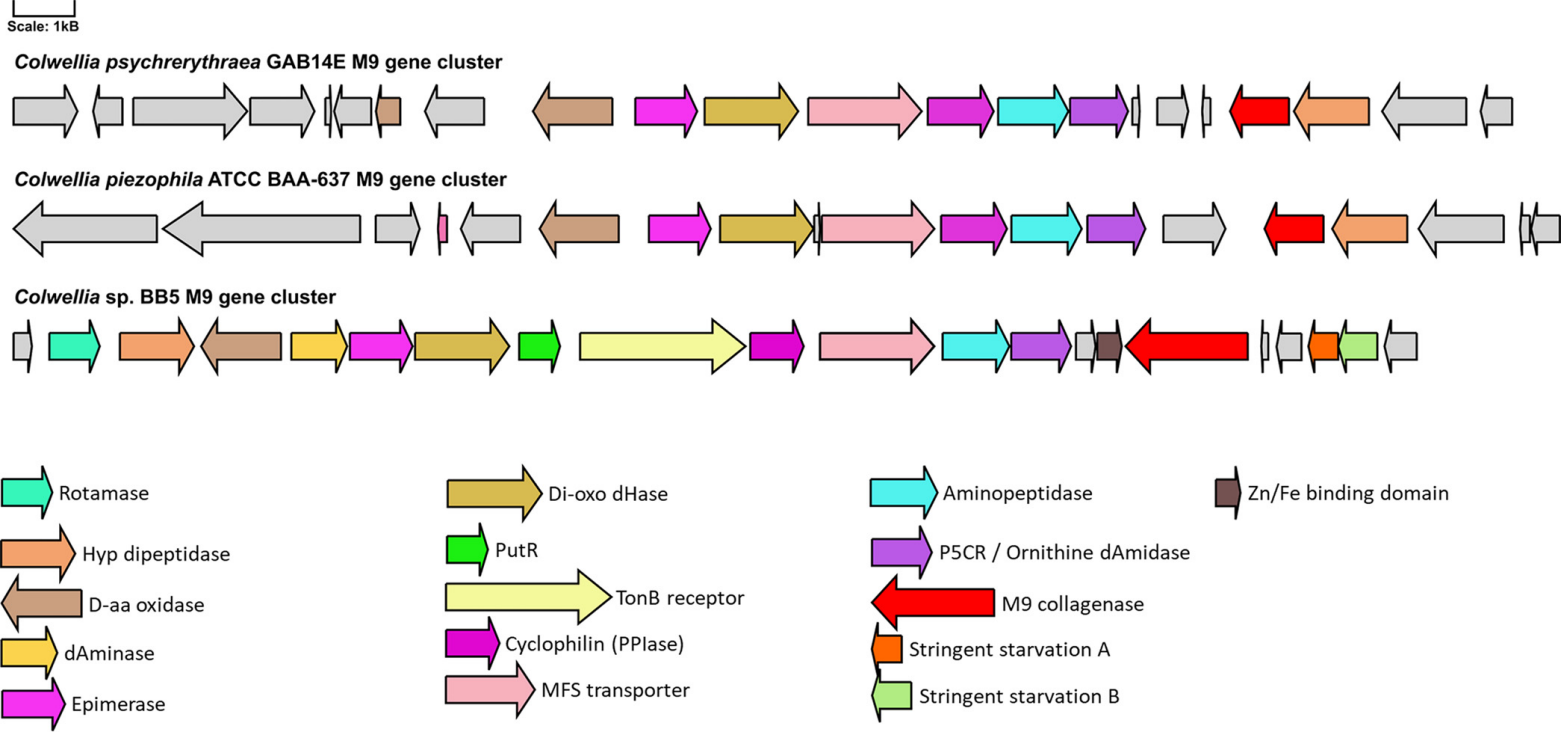
Conservation between M9 collagen degradation gene clusters in Colwellia psychrerythraea GAB14E, Colwellia piezophila ATCC BAA-637, and the MAG *Colwellia* BB5 drawn at scale. dHase, dehydrogenase; PPIase, peptidyl-prolyl *cis-trans* isomerase; Hyp, D-aa, dAminase, etc. Color coding and gene names are indicated.

To explore the conservation of this gene cluster, we retrieved 14 representative *Colwellia* genomes of marine origin from the NCBI repository (Table S4) (50–63). To minimize methodological bias, the nucleotide sequences of these genomes were likewise annotated with RAST (rapid annotation using subsystem technology) and screened for M9 collagenase using the previously established HMM profile. Twenty-two annotated M9 collagenases were identified in seven out of 14 genomes. In the genomes of Colwellia piezophila ATCC BAA-637 (57) and Colwellia psychrerythraea GAB14E (59), a gene cluster comparable to the one in MAG BB5 was identified (Fig. 6) and found to be largely conserved between the three species. The conserved core is constituted by the M9 collagenase, a d-hydroxyproline dehydrogenase, an epimerase, a secreted cyclophilin-type peptidyl-prolyl isomerase, a ketoglutaric acid dehydrogenase, an MFS transporter, an aminopeptidase, a bifunctional 1-pyrroline-5-carboxylate reductase/ornithine cyclodeaminase, and a hydroxyproline dipeptidase. BB5 additionally contains several other relevant genes, such as a PutR regulator, stringent starvation proteins A and B, a TonB-dependent receptor, Zn/Fe binding domain protein, 1-pyrroline-4-hydroxy-2-carboxylate deaminase, and an intracellular peptidyl-prolyl *cis-trans* isomerase (rotamase, PpiD).

10.1128/mSystems.01218-20.8 TABLE S4 *Colwellia* genomes used in this study for comparison to MAG BB5. Download Table S4, DOCX file, 0.03 MB. Copyright © 2021 Borchert et al. 2021 Borchert et al. This content is distributed under the terms of the Creative Commons Attribution 4.0 International license.

10.1128/mSystems.01218-20.9 TABLE S5 Quality control report of all high-quality MAGs. The figures were produced with the reassemble bins module within the MetaWRAP pipeline, which utilizes CheckM (99) for quality control. (A) Bone biofilm MAGs (BB) listed according to ascending completeness and with a depiction of presence of single-copy marker genes, heterogeneity, and contamination. (B) *Osedax* bone MAGs (OB) listed according to ascending completeness and with a depiction of presence of single-copy marker genes, heterogeneity, and contamination. Download Table S5, DOCX file, 1.0 MB. Copyright © 2021 Borchert et al. 2021 Borchert et al. This content is distributed under the terms of the Creative Commons Attribution 4.0 International license.

### Specificity of the bone microbiome.

To investigate the potential specificity of the bone microbiome in respect to its taxonomic composition and function, the generated MAGs were compared to 832 seawater MAGs generated from the Tara Oceans data sets, comprising 93 metagenomes from various locations (64). The bone microbiome was rebinned to retrieve all MAGs with >70% completion to match the Tara Oceans threshold for high-quality MAGs, resulting in 86 MAGs. The taxonomic class abundances were compared between the two data sets, and Gammaproteobacteria and Bacteroidia appeared in similar abundances (bone microbiome, 23.26% and 15.12%, and Tara Oceans, 23.56% and 13.34%, respectively), but other than those high taxonomic ranks, the data sets were different. The bone microbiome is dominated by Campylobacteria, accounting for 26.74% of the MAGs, whereas only 0.12% Campylobacteria MAGs are found in the Tara Oceans data set. In contrast, Alphaproteobacteria are better represented in the Tara Oceans data set than in the bone microbiome, 21.88% to 5.81%, respectively. The bone microbiome furthermore also contains a number of bacterial classes that are represented at low levels (1 to 5% of the MAGs), which are either not represented in the Tara Oceans data set or represented only at minuscule levels as low as 0.1 to 0.5% (Fig. S4A). The functional repertoire was compared via screening the Tara Oceans data set with the previously generated HMM profiles for enzymes potentially involved in bone degradation and calculated as ratio of enzymes per MAG. The ratios for 9 out of 12 profiled enzyme families were higher in the bone microbiome, ratios for α-*N*-acetylgalactosaminidases (COG0383) and cholesterol oxidases (COG2303) were higher in the Tara Oceans data set, and the ratio for S1 peptidases was equal between the two MAG sets (Fig. S4B).

## DISCUSSION

In this study, 59 high-quality MAGs were reconstructed from microbes colonizing bone surfaces and from symbionts of the bone-eating worm *Osedax mucofloris*. Metabolic reconstruction revealed a complex, diverse, and specialized community. Our MAGs span at least 23 bacterial families and uncover a large potential for taxonomic novelty (over 50% according to genome-based taxonomy) from species up to class level in the bone microbiome. Interestingly, only genomes of Gram-negative bacteria were reconstructed, and despite Gram-positive bacteria being widespread in the marine environment, they make up only minor portions of the metagenomes (4 to 5% of the reads affiliated with Actinobacteria and Firmicutes in the *Osedax* metagenomes and 5 to 9% in the biofilm metagenomes, respectively) (65). This is remarkable since they are known to carry out potentially relevant metabolic processes (thiotrophy, sulfidogenesis) (66, 67), are capable of dealing with low-pH conditions which are likely encountered during bone dissolution (68), and possess high capacity for the secretion of hydrolytic enzymes (69). Despite this underrepresentation of Gram-positive taxa, this study reveals the existence of a specialized bone-degrading microbiome in the marine environment and starts to explore the enzymatic activities involved in the complete demineralization of bone material. The bone microbiome is different from seawater communities and from other specialized habitats (recolonized volcanic eruption site) in its microbial composition as well as functional makeup (see Fig. S4 in the supplemental material).

### The role of *Osedax* endosymbionts in bone utilization.

Two distinct bacterial endosymbiont genomes belonging to the order Oceanospirillales have previously been sequenced, but their role in bone degradation in the marine environment remained unclear (18). The bacterial fraction of the here-sequenced *Osedax mucofloris* metagenome is made up of 7% to 22% Oceanospirillales-affiliated reads, whereas the bone surface metagenome contains only 1% to 4% reads of this order according to the performed Kaiju analysis. This relative difference confirms that the methodological approach to minimize cross-contamination was successful and that the OB-MAGs affiliated with Oceanospirillales likely represent the symbiotic community of *Osedax mucofloris* worms. Two MAGs belonging to the Oceanospirillales were identified in the *Osedax*-associated metagenome, belonging to the genera *Neptunomonas* (OB1) and *Amphritea* (OB2). Both genera are known to have an aerobic organotrophic metabolism and are also able to thrive as free-living bacteria (70). In fact, the scarce representation of Oceanospirillales in the bone surface has been reported before (30, 31) and contrasts with their character as common dwellers in the marine environment (71, 72). Although we cannot rule out that Oceanospirillales preferentially colonize bone surfaces in earlier or later stages, their preference for a symbiont life supports the notion of a casual and facultative association with *Osedax* worms, triggered by the common benefit from a sudden nutrient pulse as previously hypothesized (18, 21).

### The degradative functions within the bone microbiome. (i) Acidification via a closed sulfur biogeochemical cycle.

Free-living microbial communities must deal with similar challenges as the *Osedax* holobiont to access the nutrient-rich, collagen-made organic bone matrix and eventually the lipid-rich bone marrow by dissolving the hydroxyapatite. The association in large specialized microbial consortia may be a beneficial strategy for achieving this task. We hypothesize that sulfur-driven geomicrobiology (sulfate/thiosulfate/tetrathionate reduction and sulfide/sulfur/thiosulfate oxidation) is the major factor responsible for bone dissolution in the marine environment by free-living bacterial communities. Campylobacterales are one of the most abundant bacterial orders in the here-investigated metagenomes, in both the *Osedax-*associated metagenomes (OB) and the bone surface biofilms (BB). Campylobacterales represent the most abundant group in terms of absolute read number, although it is the second largest taxon with reconstructed MAGs (Fig. 1). Members of the Campylobacterales have previously been found to be associated with *Osedax*, albeit not as endosymbionts (21). The majority of retrieved Campylobacterales MAGs (14 in total) belong to different families of aerobic and facultative anaerobic (nitrate, manganese) sulfur-oxidizing bacteria (73, 74) (Thiovulaceae, Sulfurovaceae, and Arcobacteraceae). Other aerobic/facultatively anaerobic (nitrate) sulfur-oxidizing bacteria are also well represented in the order Beggiatoales (Gammaproteobacteria, 5 MAGs). *Beggiatoa-*like bacterial mats are commonly associated with whale falls (75), indicating a potential indifference regarding the bone type they dwell on. Sulfide oxidation produces elemental sulfur or sulfate (41, 45) while releasing protons and thereby causing a drop in pH. This acidification mechanism has been linked to bone demineralization. The dissolution of the hydroxyapatite mineral exposes the organic matrix to enzymatic degradation (30, 31). Besides thiotrophy, which seems to be a major acid-producing mechanism in the microbial community, other mechanisms might also contribute significantly. In this respect, a number of carbonic anhydrases (CAs) were annotated, which are normally housekeeping genes involved in internal pH homeostasis and other processes (76) but known to play a role in environmental acidification by *Osedax* (15). Here, the CAs were found to contain signal peptides for extracellular export (19 out of 94) and therefore could also be involved in acidification. Interestingly, 18 out of 19 identified and potentially secreted CAs belong to the α-CA family and only one member of the β-CA family was found (Fig. S2). The α-CA family is found only in Gram-negative bacteria, which is also the case here, and it is evolutionarily the youngest of the three bacterial CA families (49).

Besides a large number of SOB, eight MAGs related to SRB were identified that are affiliated with the families Desulfobulbaceae (also SOB), Desulfobacteraceae, Geopsychrobacteraceae, and Desulfovibrionaceae. Moreover, they are prevalently associated with the free-living community attached to the bone surface in this study. Sulfate, tetrathionate, or thiosulfate can serve as electron acceptors and/or donors, and gene markers for all pathways are present in the genomes (Fig. 2). Microbial sulfidogenesis on the bone surface or the surrounding sediments can feed the thiotrophic community and therefore accelerate the demineralization process. The generated sulfide is known to quickly react with iron, blackening the bone surfaces with insoluble iron sulfide (77). In our incubation experiments, blackening occurs preferentially on the epiphysis, which is also where complex white/pink microbial mats are forming over time (Fig. S1B). However, from our analysis SRB seem unable to degrade large complex molecules. This is supported by the lack of bone-degrading enzymes here investigated, such as S8/S53 peptidases, mannosidases, sialidases, fucosidases, and α-galactosidases. SRB are likely dependent on the generation of simple organic compounds produced as metabolites by fermenters or aerobic organotrophic bacteria of the wider bone microbiome. The bone dissolution driven by sulfur geomicrobiology relies on other specialized members of the community to degrade the organic matrix and to fuel the acid generation.

### (ii) Degradation of organic compounds via peptidases, glucosidases, and oxidases.

Once the inorganic hydroxyapatite is removed, an array of different enzymes is required to digest the various organic bone components. Bacteroidia appear to be especially remarkable in this respect and represent the third most abundant taxon. Eight high-quality MAGs could be reconstructed, seven of them from the bone surface metagenome. Bacteroidia, and especially the family of Flavobacteriaceae, are known to be versatile degraders of polysaccharides like agar (78), chitin (79), ulvan (80), alginate (81), carrageen (82), and cellulose and xylanose (83) and polypeptides like elastin (84), spongin (85), and others. The recently described Marinifilaceae family (86) includes isolates that are reported to present xylanase activity (87). Despite the discrepancy between abundance and reconstructed genomes, the Bacteroidia MAGs appear to be the most versatile order of the investigated MAGs in respect to their richness in bone-degrading enzymes (Fig. 4), and all were predicted to possess the gelatin hydrolysis trait (Fig. 3). They were also the only MAGs containing sialidases (COG4409) and α-galactosidases (Pfam16499) (Fig. 4). Due to this taxon-specific trait and their presence being limited to the bone surface-associated microbiome, we hypothesize that Bacteroidia play a pivotal and specialized role in the free-living community via the degradation of specific organic bone components.

Differential microbial colonization of the spongy cancellous bone tissue over the cortical compact bone has also been observed in the terrestrial environment and has been related to easier access to the red marrow (88), although a priming effect linked to the differential composition of the bone cannot be ruled out. Complex microbial mats form preferentially on the epiphysis of the long bones, and this area is normally covered with hyaline cartilage (89) which was not removed before deployment. Cartilage is a related connective tissue made of nonfibrous type II collagen and a sulfated-proteoglycan matrix rich in *N*-acetylgalactosamine and glucuronic acid residues. This would explain the abundance of α-galactosidases, *N*-acetylglucosaminidase, and glucuronidases. Moreover, other groups such as Kiritimatiellales (PVC superphylum) are known marine anaerobic saccharolytic microbes specialized in degrading sulfated polymers that we find in this environment (90).

### (iii) Collagen degradation by Gammaproteobacteria.

Peptidases and especially M9 collagenases are of special interest for the degradation of the proteinogenic compounds within bone, as they are able to degrade collagen, the main source of carbon in this environment. The class Gammaproteobacteria is comparatively enriched in these enzymes, and it is the best represented class in the data set, with 17 MAGs. Of particular interest are the MAGs affiliated with the order Enterobacterales (two MAGs of the families Kangiellaceae and one Alteromonadaceae). They possess the gelatin hydrolysis trait (Fig. 3, MAGs BB5, BB44, and OB12), have a high number of S1 and U32 peptidases, and are the only MAGs with M9 collagenases. The *Colwellia* MAG BB5 is particularly remarkable as it contains an entire gene cluster dedicated to collagen utilization (Fig. 5A). The collagen degradation gene cluster comprises at least 15 different genes, including an M9 collagenase, a PepQ proline dipeptidase, an aminopeptidase YpdF, several transporters, epimerase, isomerases, and others. The gene cluster encodes nearly the entire pathway necessary to unwind and hydrolyze triple-helical collagen, transport and uptake of collagen oligopeptides into the cell, and utilization of its main components, mainly hydroxyproline and proline, for energy production via the tricarboxylic acid (TCA) cycle and/or the urea cycle or for polyamine biosynthesis (Fig. 5B). Accessory genes for an alternative catabolic route of proline to glutamate are located elsewhere in the genome (P5CDH). This kind of functional condensation for collagen utilization has not been described before in *Colwellia* or elsewhere. Interestingly, *Colwellia* bacteria are also one partner in a dual extracellular symbiosis with sulfur-oxidizing bacteria in the mussel *Terua* sp. ‘Guadelope’, retrieved from a whale fall in the Antilles arc, and are supposedly involved in the utilization and uptake of bone components (91). A cluster of functionally related genes was found in the publicly available genomes of Colwellia piezophila and Colwellia psychrerythraea. However, the gene cluster described for MAG BB5 contains several supplementary features, like a rotamase, a 1-pyrroline-4-hydroxy-2-carboxylate deaminase, and a Zn/Fe binding domain protein potentially attributed to collagen utilization, which are absent in the published genomes (Fig. 6). Moreover, the gene cluster contains regulatory elements like the PutR regulator and stringent starvation proteins known to be activated under acid stress or amino acid starvation conditions in Escherichia coli (92). This supports our hypothesis that other members of the microbial community need to dissolve the bone calcium phosphate via acid secretion, before collagen and other organic bone compounds can be accessed. In summary, the publicly available gene clusters lack regulatory elements to switch on the collagen utilization pathway under “bone-degrading”/acidified conditions and are missing key enzymes to exploit collagen’s key components proline (rotamase missing to transverse d-proline to l-proline) and hydroxyproline (pyrroline-4-hydroxy-2-carboxylate deaminase missing that breaks down 1-pyrroline-4-hydroxy-2-carboxylate to alpha-ketoglutarate semialdehyde).

### (iv) Bone degradation—a complex microbial community effort.

**FIG 7:**
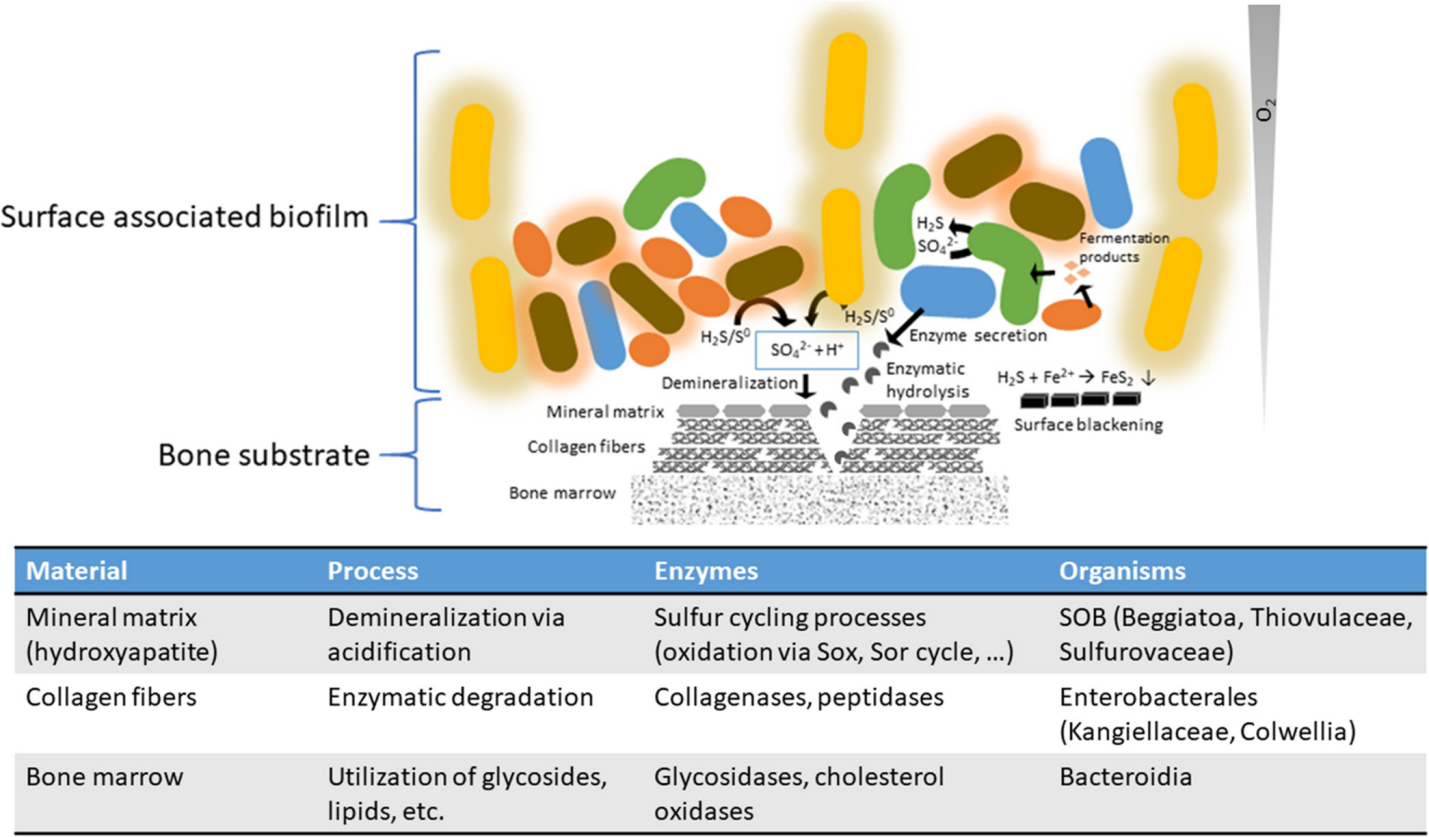
Hypothesis of the interplay in the marine bone microbiome and degradome. Sulfur-oxidizing bacteria (SOB, shown with a halo) convert elemental sulfur and H_2_S into sulfate and protons that lead to an acidification and therefore bone demineralization. Sulfate-reducing (SRB, green) and sulfur-disproportioning bacteria produce H_2_S from sulfate. Enterobacterales and others, especially Gammaproteobacteria, secrete collagenases to degrade collagen. Bacteroidia and other bacteria secret glycosidases and other enzymes to hydrolyze the organic bone components (glycosides, esters, lipids). This exemplifies a bone demineralization loop that fuels itself as long as sulfur is available and degrades the organic bone components in the process.

The marine bone microbiome is a complex assemblage of various bacterial classes that requires the synergistic action of many different interwoven enzymatic reactions to access the recalcitrant bone material for its nutritional resources. Based on metagenomic predictions, we envision the following scenario of these complex processes (Fig. 7). The primary requirement in utilizing organic bone compounds is likely the dissolution of mineralized calcium phosphate (hydroxyapatite) by acidification, which can potentially be performed via proton release by a versatile community of sulfur-oxidizing (SOB) *Gammaproteobacteria* (mainly *Beggiatoa*-like), Campylobacterales (*Sulfurimonas*, *Sulfurospirillum*, *Sulfurovum*), Desulfobulbales, and Alphaproteobacteria. This acidification via thiotrophy may be fueled by sulfur-reducing bacteria (SRB), like Desulfobacteraceae, Geopsychrobacteraceae, and *Pseudodesulfovibrio*, creating a sulfur biogeochemical loop between SRB and SOB. Once the organic compounds (collagen, fatty acids, proteins, and peptidoglycans) are accessible, the Bacteroidia (Flavobacteriaceae and Marinifilaceae) and Gammaproteobacteria (Alteromonadaceae and Kangiellaceae) may become the main protagonists. These Bacteroidia are especially rich in bone-degrading enzymes, but importantly, the Gammaproteobacteria are the only members identified with M9 collagenases, and one genome identified as *Colwellia* contains an entire gene cluster dedicated to collagen degradation (Fig. 5). Here, we disentangled the potential functional roles of specialized members of the bone-degrading microbial community, which together make bone-derived nutrients accessible—not only to themselves but also to generalists within the bone microbiome. We posit that Flavobacteriales and Enterobacterales are the most promising candidates for novel enzyme discovery, as they display the most versatile sets of bone-degrading enzymes.

## MATERIALS AND METHODS

### Sample collection.

Bone material after manual meat deboning was kindly provided by a local slaughterhouse operated by Norilia (Norway). Since deboning does not completely eliminate the animal tissue attached to the bone, some remains were still present. Therefore, in order to avoid bacterial colonization and decomposition, all bone material was kept at −20°C until deployment. Four sets of turkey thigh bones and one bovine lower leg bone were placed in a crab trap and deposited at the bottom of Byfjorden (60.397093N; 5.301293E) close to Bergen, Norway, at a depth of 69 m and approximately 150 m offshore in May 2016, incubated for 9 months, and retrieved using a small ROV (Table 1). The material was transported to the lab in Styrofoam boxes either for processing within 2 h (bone surfaces) or for prolonged incubation in seawater aquaria and subsequent dissection of *Osedax* worms. The meatless bone surfaces were scraped with a sterile scalpel for microorganisms, and *Osedax mucofloris* specimens were extracted from the bone using sterile scissors and forceps. Their root tissue was dissected from the body, rinsed in sterile 70% (vol/vol) seawater, preserved in storage solution (700 g/liter ammonium sulfate, 20 mM sodium citrate, and 25 mM EDTA, pH 5.2), and stored at −70°C until further processing.

### DNA extraction and sequencing.

DNA was extracted from 10- to 50-mg samples of either scraped biofilm or *Osedax* root tissue, using the Qiagen AllPrep DNA/RNA minikit according to the manufacturer’s instructions with cell lysis by a bead beating step in Lysing Matrix E tubes (MP Biomedicals) in a FastPrep homogenizer (MP Biomedicals) with a single cycle of 30 s at a speed of 5,500 rpm. The obtained DNA was quantified and quality controlled using a NanoDrop2000 (ThermoFisher Scientific) and a Qubit fluorometer 3.0 (ThermoFisher Scientific). The obtained DNA concentrations ranged from 11.9 ng/μl to 166 ng/μl according to Qubit readings. The DNA libraries were prepared using the Nextera DNA library prep kit according to the manufacturer’s guidelines. Fifty nanograms of DNA was used for the preparations. In brief, the DNA was fragmented by the Nextera transposome at 55°C for 5 min and barcoded adapters were added in a 5-cycle PCR amplification. The resulting libraries, including ∼140 bp of adapter, had an average fragment size of 436 bp (±112 bp) and were sequenced on an Illumina HiSeq4000 platform (150-bp paired-end reads) at the Institute of Clinical Molecular Biology (IKMB), Kiel University, Germany.

### Metagenomic read profiling.

Illumina raw reads were quality trimmed and adapters were removed with Trimmomatic version 0.36 (93). The quality-filtered reads were used individually and combined with respect to their sample source (either *Osedax*-associated or bone surface biofilms) to profile the taxonomic origin of the reads with Kaiju (35).

### Metagenomic assembly, binning, taxonomic identification, ORF prediction, and annotation.

For each sample type (*Osedax mucofloris* and bone surface biofilm communities), the quality-filtered metagenomic reads (Trimmomatic version 0.36) were coassembled with SPAdes v3.12 (94) for kmers 21, 33, 55, 77, and 99, with the metaSPAdes-assembler option enabled (see Table S1 in the supplemental material for read counts). Binning was conducted on the resulting assemblies using the MetaWRAP pipeline (version 1.0.1) (95). This pipeline combines initially three different binning methods, CONCOCT (96), MaxBin2.0 (97), and metaBAT2 (98), to generate MAGs. In the next step, all MAGs are combined in different MAG sets and a bin_refinement module is utilized to choose the best version of each MAG according to the desired minimum completion and maximum contamination levels. We considered only high-quality MAGs with >90% completeness and <10% redundancy for further analyses. CheckM was used for quality assessment of the assembled genomes (99), and GTDB-Tk version 0.1.3 (36) was used for taxonomic identification, coupled with an estimate of relative evolutionary divergence (RED) to their next common ancestor. RED is a normalization method to assign taxonomic ranks according to lineage-specific rates of evolution, based on branch lengths and internal nodes in relation to the last common ancestor calculated by GTDB-Tk. Open reading frames (ORFs) of the obtained MAGs were predicted with Prodigal version 2.6.3 (100). Predicted ORFs were annotated using eggNOG-mapper v1 (101) with eggNOG orthology data version 4.5 (102). Additionally, the MAGs were annotated and metabolic models were calculated using the RAST (rapid annotation using subsystem technology) server (103, 104). The MAGs were further investigated for the presence or absence of major metabolic pathways and phenotypic microbial traits based on their genomic sequences using MEBS (multigenomic entropy-based score, version 1.2) (37) and Traitar (40). MEBS is a software package used here to detect genes related to sulfur metabolism; this was done by providing protein fasta files of the high-quality MAGs that are annotated by MEBS with InterProScan (105) and are then searched with HMM profiles for genes related to sulfur metabolism. The sulfur metabolism-related genes investigated by MEBS are based primarily on the MetaCyc database (106). Traitar is a software package that can predict 67 phenotypic traits from a genome sequence. In brief, the analysis is based on known phenotypic traits of 234 bacterial species and infers from their genome Pfam families that are either present or absent in a specific trait. In this paper, the traits gelatin hydrolysis and H_2_S production are of interest and these are based on the presence of 70 and 43 and absence of 51 and 22 Pfam families, respectively. Phylogenomic trees were calculated with FastTree (107) as maximum likelihood trees and visualized with iTOL (108, 109), and heatmaps were visualized with Heatmapper (110). Gene cluster maps were drawn with Gene Graphics (111). Signal peptides were predicted with the SignalP-5.0 server using nucleotide sequences to predict the presence of Sec/SPI, Tat/SPI, and Sec/SPII signal peptides in a given sequence (112).

### Enzyme profiling.

Based on the organic composition of bone matrix, we hypothesized 12 enzyme families to be necessary for its degradation. Accordingly, the following enzymes were selected for in-depth studies: (i) M9 collagenases (pfam01752), S1 peptidases (COG0265), S8/S53 peptidases (pfam00082), and U32 proteases (COG0826), which hydrolyze peptide bonds in collagen and glycoproteins; (ii) sialidases (COG4409), β-d-glucuronidases (COG3250), β-*N*-acetyl-d-glucosaminidases (COG1472), α-*N*-acetylgalactosaminidases (COG0673), α-galactosidases (pfam16499), fucosidases (COG3669), and mannosidases (COG0383), which cleave glycosidic linkages; and (iii) cholesterol oxidases (COG2303), which degrade lipids such as cholesterol. One reference database for each of these families was generated using the NCBI repository, based on sequences from 287 M9 collagenases, 4,453 S1 peptidases, 3,237 S8/S53 peptidases, 3,653 U32 proteases, and 267 COG4409, 873 COG3250, 1,274 COG1472, 6,140 COG0673, 279 COG3669, 206 COG0383, and 1,119 COG2303 enzymes. The databases included the closest protein homologs of all protein families of interest for bone degradation, and at least one representative sequence from all taxonomic groups (containing such enzymes) was represented. The reference databases were used to generate hidden Markov model (HMM) profiles for each enzyme family with HMMer version 3.1b1 (113) using the *hmmbuild* option after an alignment of each sequence set was built with Clustal W version 2.1 (114). The MAGs were screened for the 12 enzyme families of interest using the generated HMM profiles using HMMer version 3.1b1 with the *hmmsearch* option and a bitscore threshold of 100.

### Tara Oceans comparison.

This comparison is based on the work of Delmont et al. (2018), who binned 93 metagenomes generated from the Tara Oceans project (64). The metagenomes represent 61 surface water samples and 32 samples from the deep chlorophyll maximum layer of the water column. They generated 957 nonredundant high-quality bacterial, archaeal, and eukaryotic genomes. The 957 MAGs were here reanalyzed with GTDB-Tk and the generated HMM profiles as previously described. Eight hundred thirty-two MAGs were identified as of bacterial origin and included in the comparison. The Tara Oceans MAGs were generated with a quality threshold of >70% completion; therefore, the bone metagenomes were rebinned (>70% completion, <10% redundancy) for better comparison to avoid bias due to the higher threshold used for functional analysis in other parts of this paper.

### Data availability.

The raw sequencing reads have been deposited in the Sequence Read Archive (SRA) of NCBI under the BioProject ID PRJNA606180 and with the BioSample accession numbers SAMN14086998 (A5), SAMN14087000 (A9), SAMN14087001 (A9n), SAMN14087003 (B4), SAMN14087005 (D1), SAMN14087006 (D2), SAMN14087007 (I1), and SAMN14087008 (I3).

The 59 high-quality MAGs analyzed in this study were deposited in the NCBI database as well, as part of the BioProject ID PRJNA606180; the BioSample accession numbers are SAMN16086327 to SAMN16086385 (biofilm MAGs 1 to 44, SAMN16086327 to SAMN16086370, and *Osedax* MAGs 1 to 15, SAMN16086371 to SAMN16086385).
