# Supplementary figures and images for: Deciphering a Marine Bone-Degrading Microbiome Reveals a Complex Community Effort
Source: mSystems. 2021 Feb 9;6(1):e01218-20. doi: 10.1128/mSystems.01218-20 (PMC7883544; doi:10.1128/mSystems.01218-20)

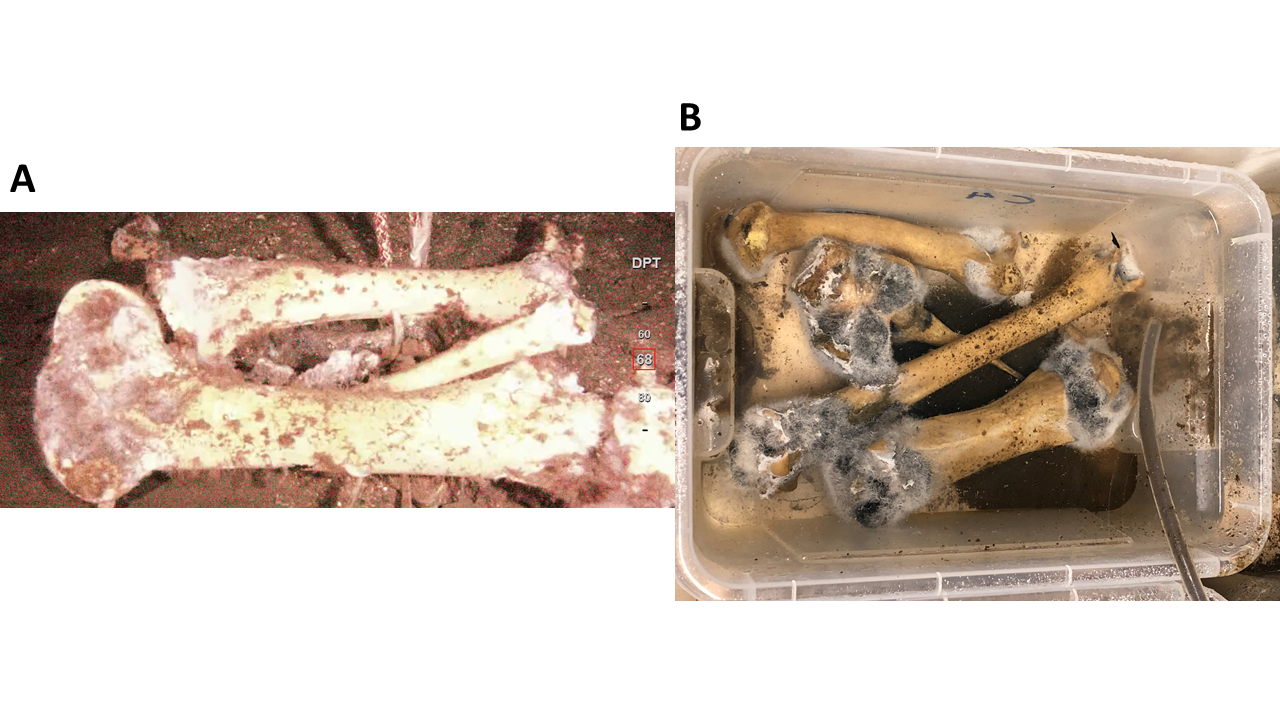

Supplement: FIG S1 [file mSystems.01218-20-sf001.tif]

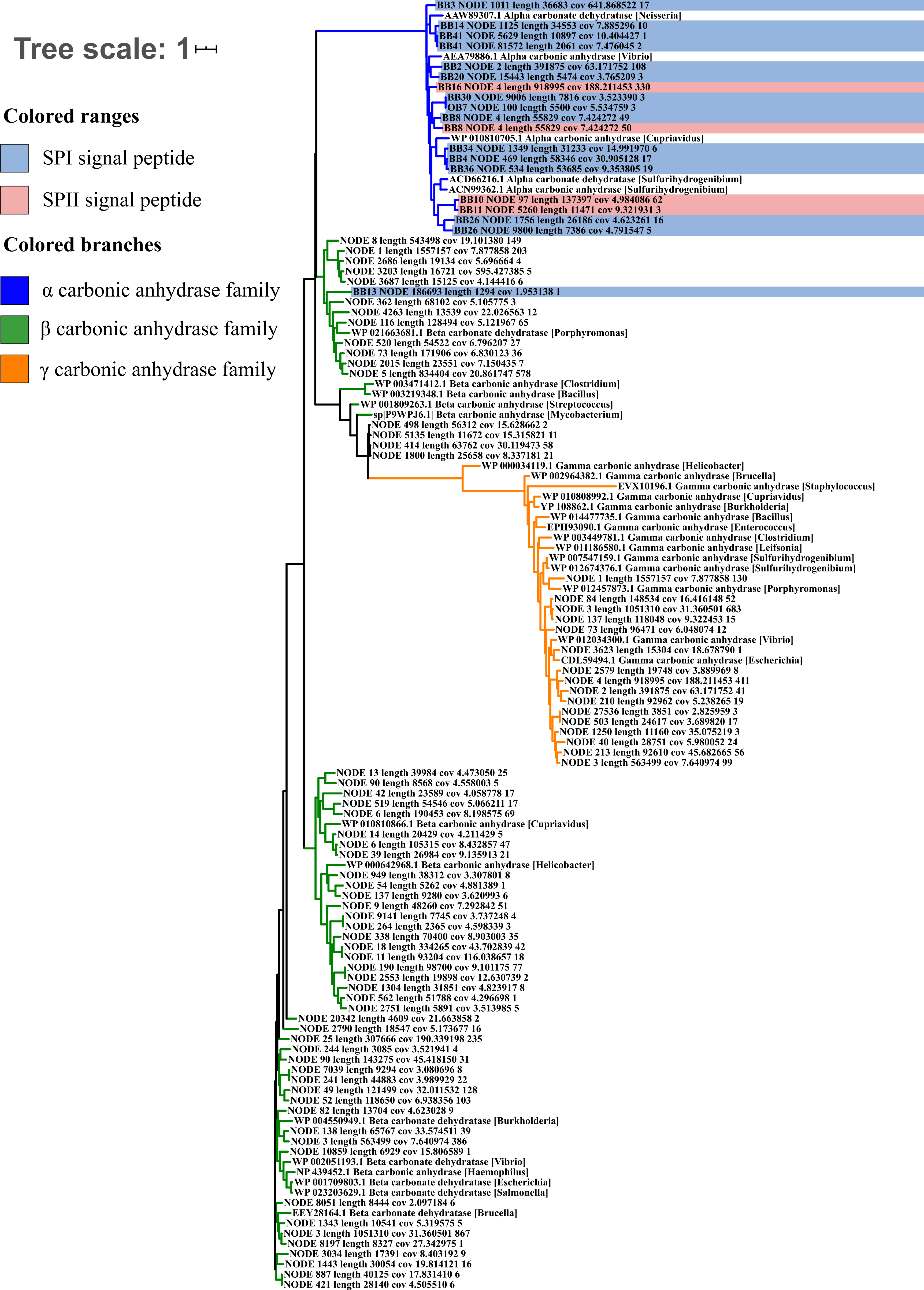

Supplement: FIG S2 [file mSystems.01218-20-sf002.tif]

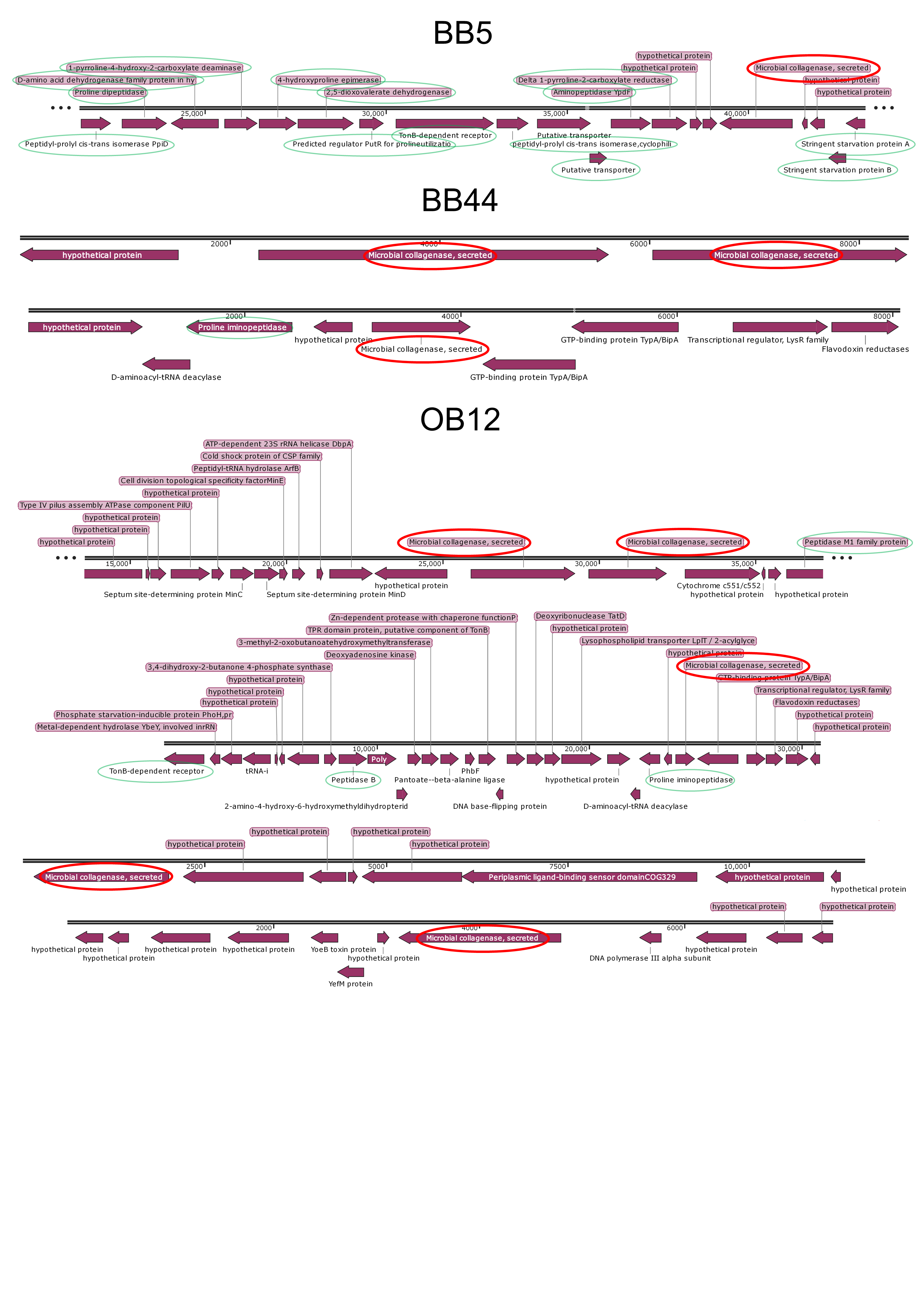

Supplement: FIG S3 [file mSystems.01218-20-sf003.tif]

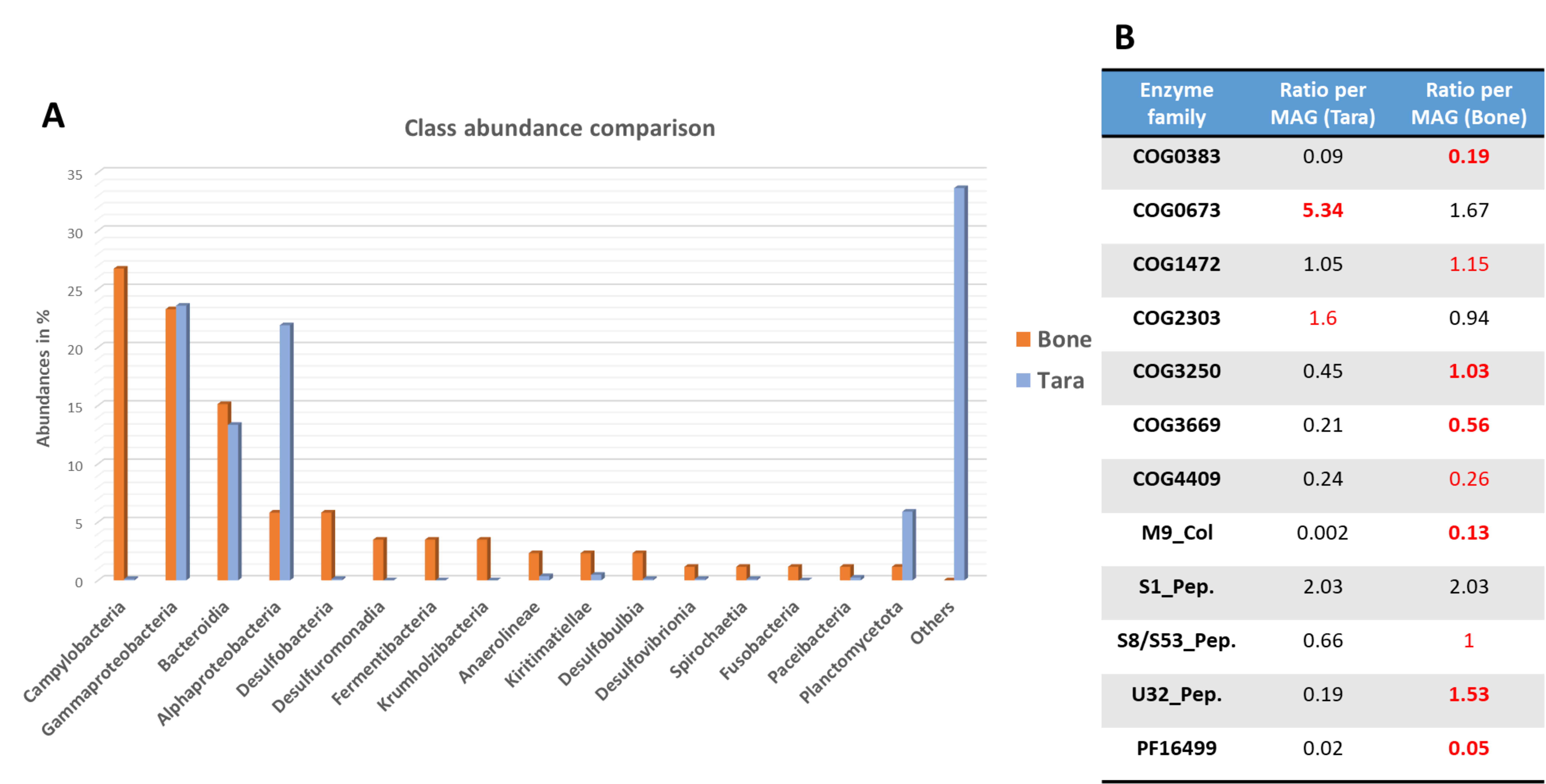

Supplement: FIG S4 [file mSystems.01218-20-sf004.tif]
