# Supplementary material for: Deciphering a Marine Bone-Degrading Microbiome Reveals a Complex Community Effort
Source: mSystems. 2021 Feb 9;6(1):e01218-20. doi: 10.1128/mSystems.01218-20 (PMC7883544; doi:10.1128/mSystems.01218-20)
Supplement: TABLE S1 [file mSystems.01218-20-st001.docx]

| **Taxonomic distribution of raw reads (Kaiju)** | Archaea [%] | 0.40 | 0.40 | 0.40 | 0.40 | 0.40 | 0.40 | 0.40 | 0.40 |
| --- | --- | --- | --- | --- | --- | --- | --- | --- | --- |
|  | Eukaryota (fungi) [%] | 15 (4) | 34 (9) | 34 (9) | 34 (9) | 34 (9) | 34 (9) | 34 (9) | 34 (9) |
|  | Bacteria [%] | 76 | 45 | 45 | 47 | 94 | 92 | 95 | 93 |
| **Combined statistics** | N50 | 1115 | | | | 1293 | | | |
|  | contigs | 309.5k | | | | 1022k | | | |
|  | size [Mb] | 342.7 | | | | 1220 | | | |
| **Individual statistics** | largest contig | 1645k | 156k | 299k | 137k | 918k | 1234k | 1105k | 195k |
|  | N50 | 1175 | 1007 | 926 | 910 | 2543 | 1134 | 1336 | 1136 |
|  | contigs | 185.1k | 169k | 90.8k | 132.7k | 121.8k | 156.9k | 440.8k | 227.9k |
|  | size [Mb] | 214.6 | 170.9 | 88.6 | 120.7 | 192.9 | 175.2 | 534.3 | 249.1 |
| **Reads** | dereplicated [million] | 13.32 | 12.8 | 5.12 | 8.86 | 12.16 | 15.67 | 26.07 | 15.48 |
|  | filtered [million] | 18.75 | 18.71 | 6.76 | 12.65 | 20.59 | 32.5 | 31.96 | 29.29 |
|  | raw [million] | 24.8 | 24.9 | 14.4 | 19.2 | 24.1 | 35.8 | 35.9 | 32.3 |
|  | | **A5** | **A9** | **A9n** | **B4** | **D1** | **D2** | **I1** | **I3** |
