# Supplementary material for: Deciphering a Marine Bone-Degrading Microbiome Reveals a Complex Community Effort
Source: mSystems. 2021 Feb 9;6(1):e01218-20. doi: 10.1128/mSystems.01218-20 (PMC7883544; doi:10.1128/mSystems.01218-20)
Supplement: TABLE S2 [file mSystems.01218-20-st002.docx]

| **MAG** | **Phylum** | **Class** | **Order** | **Family** | **Genus** |
| --- | --- | --- | --- | --- | --- |
| BB1 | Planctomycetota* | - | - | - | - |
| BB2 | Proteobacteria | γ-proteobacteria | Beggiatoales* | - | - |
| BB3 | Proteobacteria | γ-proteobacteria | Beggiatoales | Beggiatoaceae* | - |
| BB4 | Proteobacteria | γ-proteobacteria* | - | - | - |
| BB5 | Proteobacteria | γ-proteobacteria | Enterobacterales | Alteromonadaceae | Colwellia |
| BB6 | Proteobacteria | α-proteobacteria | Rhizobiales* | - | - |
| BB7 | Spirochaetota | Spirochaetia | Spirochaetales | Spirochaetaceae* | - |
| BB8 | Campylobacterota | Camylobacteria | Campylobacterales | - | - |
| BB9 | Campylobacterota | Camylobacteria | Campylobacterales | Sulfurovaceae | Sulfurovum |
| BB10 | Campylobacterota | Camylobacteria | Campylobacterales | Thiovulaceae* | - |
| BB11 | Campylobacterota | Camylobacteria | Campylobacterales | Thiovulaceae | Sulfurimonas |
| BB12 | Fermentibacterota | Fermentibacteria | Fermentibacterales | Fermentibacteraceae* | - |
| BB13 | Desulfobacterota | Desulfobulbia | Desulfobulbales | Desulfobulbaceae* | - |
| BB14 | Campylobacterota | Camylobacteria | Campylobacterales | Sulfurovaceae* | - |
| BB15 | Campylobacterota | Camylobacteria | Campylobacterales | Sulfurospirillaceae | Sulfurospirillum |
| BB16 | Proteobacteria | γ-proteobacteria | Beggiatoales | Beggiatoaceae | Marithrix* |
| BB17 | Bacteroidota | Bacteroidia | Flavobacteriales | Ichthyobacteriaceae* | - |
| BB18 | Krumholzibacteriota | Krumholzibacteria* | - | - | - |
| BB19 | Proteobacteria | γ-proteobacteria | Pseudomonadales | Hahellaceae* | - |
| BB20 | Proteobacteria | γ-proteobacteria | Beggiatoales* | - | - |
| BB21 | Desulfuromonadota | Desulfuromonadia | Desulfuromonadales | Geopsychrobacteraceae | Desulfuromusa |
| BB22 | Bacteroidota | Bacteroidia | Bacteroidales* | - | - |
| BB23 | Bacteroidota | Bacteroidia | Flavobacteriales | Flavobacteriaceae | Winogradskyella |
| BB24 | Bacteroidota | Bacteroidia | Flavobacteriales | Flavobacteriaceae* | - |
| BB25 | Desulfuromonadota | Desulfuromonadia | Desulfuromonadales | Geopsychrobacteraceae | Desulfuromusa |
| BB26 | Campylobacterota | Camylobacteria | Campylobacterales | Thiovulaceae* | - |
| BB27 | Desulfuromonadota | Desulfuromonadia | Desulfuromonadales | Geopsychrobacteraceae* | - |
| BB28 | Campylobacterota | Camylobacteria | Campylobacterales | Arcobacteraceae* | - |
| BB29 | Bacteroidota | Bacteroidia | Bacteroidales | Marinifilaceae | - |
| BB30 | Campylobacterota | Camylobacteria | Campylobacterales | Arcobacteraceae | Arcobacter |
| BB31 | Proteobacteria | γ-proteobacteria | Beggiatoales* | - | - |
| BB32 | Proteobacteria | γ-proteobacteria | Xanthomonadales | Marinicellaceae* | - |
| BB33 | Proteobacteria | α-proteobacteria | Rhodobacterales | Rhodobacteraceae | Lentibacter* |
| BB34 | Proteobacteria | γ-proteobacteria* | - | - | - |
| BB35 | Bacteroidota | Bacteroidia | Flavobacteriales | Flavobacteriaceae | - |
| BB36 | Proteobacteria | γ-proteobacteria* |  |  |  |
| BB37 | Proteobacteria | γ-proteobacteria | Pseudomonadales | Halieaceae* | - |
| BB38 | Chloroflexota | Anaerolineae | Anearolineales* | - | - |
| BB39 | Krumholzibacteriota | Krumholzibacteria* | - | - | - |
| BB40 | Desulfobacterota | Desulfobacteria | Desulfobacterales | Desulfobacteraceae | - |
| BB41 | Campylobacterota | Camylobacteria | Campylobacterales | Sulfurovaceae* | - |
| BB42 | Bacteroidota | Bacteroidia | Flavobacteriales | Flavobacteriaceae | Maribacter* |
| BB43 | Verrucomicrobiota | Kiritimatiellae | Kiritimatiellales* | - | - |
| BB44 | Proteobacteria | γ-proteobacteria | Enterobacterales | Kangiellaceae* | - |
| OB1 | Proteobacteria | γ-proteobacteria | Pseudomonadales | Nitrincolaceae | Neptunomonas |
| OB2 | Proteobacteria | γ-proteobacteria | Pseudomonadales | Nitrincolaceae | Amphritea |
| OB3 | Desulfobacterota | Desulfovibrionia | Desulfovibrionales | Desulfovibrionaceae | Pseudodesulfovibrio |
| OB4 | Proteobacteria | α-proteobacteria | Sphingomonadales | Emcibacteraceae* | - |
| OB5 | Campylobacterota | Camylobacteria | Campylobacterales | Thiovulaceae | Sulfurimonas |
| OB6 | Proteobacteria | γ-proteobacteria* | - | - | - |
| OB7 | Campylobacterota | Camylobacteria | Campylobacterales | Arcobacteraceae | Arcobacter |
| OB8 | Campylobacterota | Camylobacteria | Campylobacterales | Thiovulaceae | Sulfurimonas |
| OB9 | Proteobacteria | α-proteobacteria | Rhodobacterales | Rhodobacteraceae | - |
| OB10 | Verrucomicrobiota | Kiritimatiellae | Kiritimatiellales* | - | - |
| OB11 | Campylobacterota | Camylobacteria | Campylobacterales | Sulfurospirillaceae | Sulfurospirillum |
| OB12 | Proteobacteria | γ-proteobacteria | Enterobacterales | Kangiellaceae* | - |
| OB13 | Bacteroidota | Bacteroidia | Bacteroidales | Marinifilaceae | Labilibaculum* |
| OB14 | Desulfobacterota | Desulfobacteria | Desulfobacterales | Desulfobacteraceae | - |
| OB15 | Proteobacteria | α-proteobacteria* | - | - | - |
