# Supplementary material for: Deciphering a Marine Bone-Degrading Microbiome Reveals a Complex Community Effort
Source: mSystems. 2021 Feb 9;6(1):e01218-20. doi: 10.1128/mSystems.01218-20 (PMC7883544; doi:10.1128/mSystems.01218-20)
Supplement: TABLE S3 [file mSystems.01218-20-st003.docx]

| **MAG** | **Genome size [Mbp]** | **Longest contig [kbp]** | **N50 [kbp]** | **No. of contigs** | **Predicted genes** | **GC content [%]** | **No. of bone-degrading**  **Enzymes** |
| --- | --- | --- | --- | --- | --- | --- | --- |
| BB1 | 4.68 | 155 | 429 | 38 | 3723 | 44.50 | 24 |
| BB2 | 4.18 | 853 | 248 | 108 | 3538 | 44.10 | 13 |
| BB3 | 4.65 | 75 | 23 | 362 | 4126 | 37.70 | 11 |
| BB4 | 5.18 | 1.050 | 198 | 68 | 4705 | 38.80 | 13 |
| BB5 | 3.26 | 82 | 13 | 395 | 3018 | 36.10 | 16 |
| BB6 | 2.77 | 72 | 19 | 244 | 2783 | 40.10 | 10 |
| BB7 | 4.87 | 78 | 15 | 489 | 4636 | 40.10 | 22 |
| BB8 | 3.08 | 89 | 18 | 413 | 3332 | 32.90 | 11 |
| BB9 | 2.16 | 126 | 36 | 159 | 2259 | 36.80 | 5 |
| BB10 | 3.01 | 137 | 35 | 142 | 2950 | 36.20 | 11 |
| BB11 | 2.39 | 21 | 3 | 809 | 2958 | 35.40 | 6 |
| BB12 | 3.24 | 329 | 141 | 127 | 2929 | 45.10 | 10 |
| BB13 | 4.24 | 16 | 3 | 1688 | 4778 | 48 | 16 |
| BB14 | 2.32 | 74 | 33 | 103 | 2265 | 34.20 | 8 |
| BB15 | 2.68 | 415 | 191 | 33 | 2695 | 30.70 | 5 |
| BB16 | 3.09 | 481 | 237 | 22 | 2867 | 34.10 | 13 |
| BB17 | 3.64 | 81 | 16 | 327 | 3171 | 32.30 | 13 |
| BB18 | 4.43 | 267 | 114 | 176 | 3652 | 52.60 | 23 |
| BB19 | 4.07 | 273 | 132 | 88 | 3778 | 41.30 | 13 |
| BB20 | 4.49 | 73 | 19 | 338 | 3725 | 37.90 | 13 |
| BB21 | 2.89 | 145 | 51 | 186 | 2812 | 44.40 | 8 |
| BB22 | 5.23 | 124 | 28 | 294 | 4210 | 34.20 | 32 |
| BB23 | 3.72 | 128 | 30 | 190 | 3427 | 23.40 | 9 |
| BB24 | 4.64 | 57 | 16 | 582 | 4438 | 31.30 | 58 |
| BB25 | 3.31 | 211 | 67 | 118 | 3069 | 44.30 | 8 |
| BB26 | 3.59 | 77 | 23 | 242 | 3547 | 44.60 | 8 |
| BB27 | 2.58 | 39 | 9 | 514 | 2602 | 43.10 | 9 |
| BB28 | 3.02 | 103 | 27 | 187 | 3056 | 26.30 | 4 |
| BB29 | 4.16 | 122 | 21 | 304 | 3375 | 34.40 | 21 |
| BB30 | 1.99 | 35 | 9 | 292 | 2109 | 25.80 | 4 |
| BB31 | 5.34 | 141 | 35 | 263 | 4342 | 45.30 | 15 |
| BB32 | 3.62 | 326 | 117 | 52 | 3061 | 35.20 | 14 |
| BB33 | 3.25 | 137 | 77 | 74 | 3319 | 56.80 | 9 |
| BB34 | 4.87 | 115 | 31 | 302 | 4339 | 38.20 | 9 |
| BB35 | 3.13 | 1.080 | 834 | 7 | 2773 | 31.80 | 12 |
| BB36 | 4.29 | 149 | 54 | 138 | 3907 | 39.10 | 9 |
| BB37 | 3.94 | 120 | 29 | 353 | 3831 | 51.50 | 11 |
| BB38 | 3.62 | 242 | 62 | 144 | 3260 | 44.20 | 14 |
| BB39 | 4.06 | 50 | 7 | 814 | 3832 | 59.10 | 17 |
| BB40 | 6.73 | 296 | 113 | 106 | 5886 | 45.10 | 0 |
| BB41 | 2.54 | 64 | 13 | 288 | 2631 | 32.90 | 7 |
| BB42 | 3.59 | 118 | 30 | 186 | 3352 | 33.10 | 23 |
| BB43 | 3.69 | 317 | 19 | 330 | 3767 | 49.10 | 10 |
| BB44 | 4.03 | 21 | 5 | 985 | 4077 | 40.20 | 17 |
| OB1 | 4.21 | 150 | 53 | 143 | 3949 | 43.10 | 13 |
| OB2 | 4.15 | 563 | 111 | 53 | 3804 | 47.20 | 14 |
| OB3 | 3.96 | 2.090 | 2.090 | 150 | 3665 | 48.70 | 16 |
| OB4 | 2.73 | 66.9 | 15 | 778 | 2962 | 38.90 | 4 |
| OB5 | 2.05 | 107 | 50 | 81 | 2113 | 31.90 | 5 |
| OB6 | 3.88 | 180 | 68 | 100 | 3643 | 39.10 | 11 |
| OB7 | 1.78 | 24 | 5 | 586 | 1916 | 26.10 | 2 |
| OB8 | 2.92 | 106 | 17 | 269 | 3159 | 31.60 | 7 |
| OB9 | 3.13 | 432 | 190 | 55 | 3181 | 55.10 | 6 |
| OB10 | 3 | 55 | 14 | 316 | 2910 | 48.60 | 10 |
| OB11 | 2.69 | 324 | 101 | 212 | 2676 | 30.50 | 5 |
| OB12 | 4.68 | 59 | 21 | 361 | 4128 | 39.90 | 22 |
| OB13 | 6.74 | 32 | 7 | 1266 | 5928 | 34.40 | 82 |
| OB14 | 6.81 | 436 | 121 | 365 | 6165 | 44.90 | 17 |
| OB15 | 2.25 | 20 | 4 | 800 | 2345 | 36.10 | 13 |
