# Supplementary material for: Deciphering a Marine Bone-Degrading Microbiome Reveals a Complex Community Effort
Source: mSystems. 2021 Feb 9;6(1):e01218-20. doi: 10.1128/mSystems.01218-20 (PMC7883544; doi:10.1128/mSystems.01218-20)
Supplement: TABLE S4 [file mSystems.01218-20-st004.docx]

| **Name** | **NCBI BioProject accession number** | **Isolation source** | **Number of M9 collagenases** | **Reference** |
| --- | --- | --- | --- | --- |
| *Colwellia piezophila* | PRJNA182419 | Deep-sea sediment | 2 | (57) |
| *Colwellia psychrerythraea* | PRJNA258170 | *Terua* mussel | 11 | (59) |
| *Colwellia hornerae* | PRJNA516280 | Arctic sea ice | 0 | (50, 51) |
| *Colwellia demingiae* | PRJNA516284 | Arctic sea ice | 1 | (50) |
| Candidatus *Colwellia aromaticivorans* | PRJNA478776 | Microcosm experiments with oil in seawater | 0 | (52) |
| *Colwellia echini* | PRJNA420580 | Sea urchin | 0 | (53) |
| *Colwellia beringensis* | PRJNA378583 | Marine sediment, Bering Sea | 1 | (62) |
| *Colwellia agarivorans* | PRJNA371543 | Coastal sea water | 0 | (60) |
| *Colwellia marinimaniae* | PRJDB5767 | Amphipod from Challenger Deep | 4 | (56) |
| *Colwellia sediminilitoris* | PRJNA381102 | Tidal flat, South Sea, South Korea | 0 | (58) |
| *Colwellia polaris* | PRJNA380006 | Arctic sea ice | 0 | (63) |
| *Colwellia mytili* | PRJNA381102 | Mussel *Mytilus edulis* | 2 | (55) |
| *Colwellia aestuarii* | PRJNA371561 | Tidal flat Korea | 0 | (54) |
| *Colwellia chukchiensis* | PRJNA380006 | Arctic ocean | 1 | (61) |
