# Supplementary material for: Deciphering a Marine Bone-Degrading Microbiome Reveals a Complex Community Effort
Source: mSystems. 2021 Feb 9;6(1):e01218-20. doi: 10.1128/mSystems.01218-20 (PMC7883544; doi:10.1128/mSystems.01218-20)
Supplement: TABLE S5 [file mSystems.01218-20-st005.docx]

1. Quality control based on CheckM with single copy marker genes of bone biofilm (BB) reconstructed MAGs.


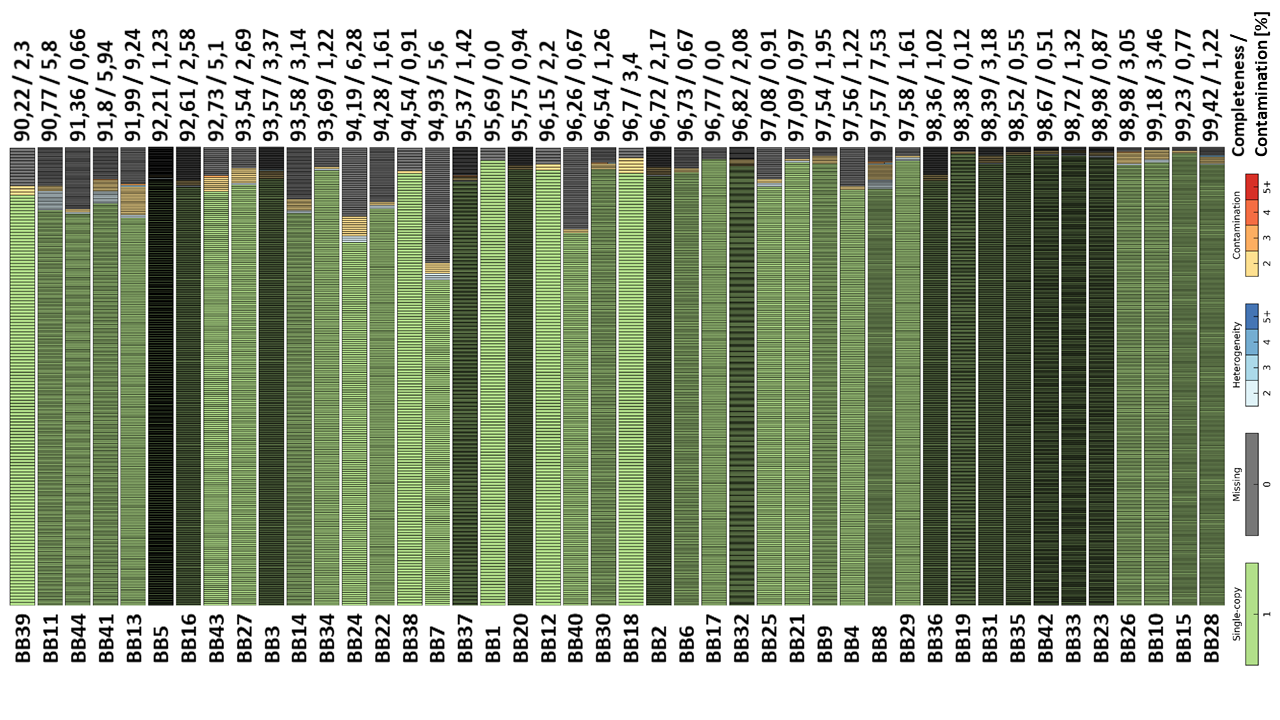


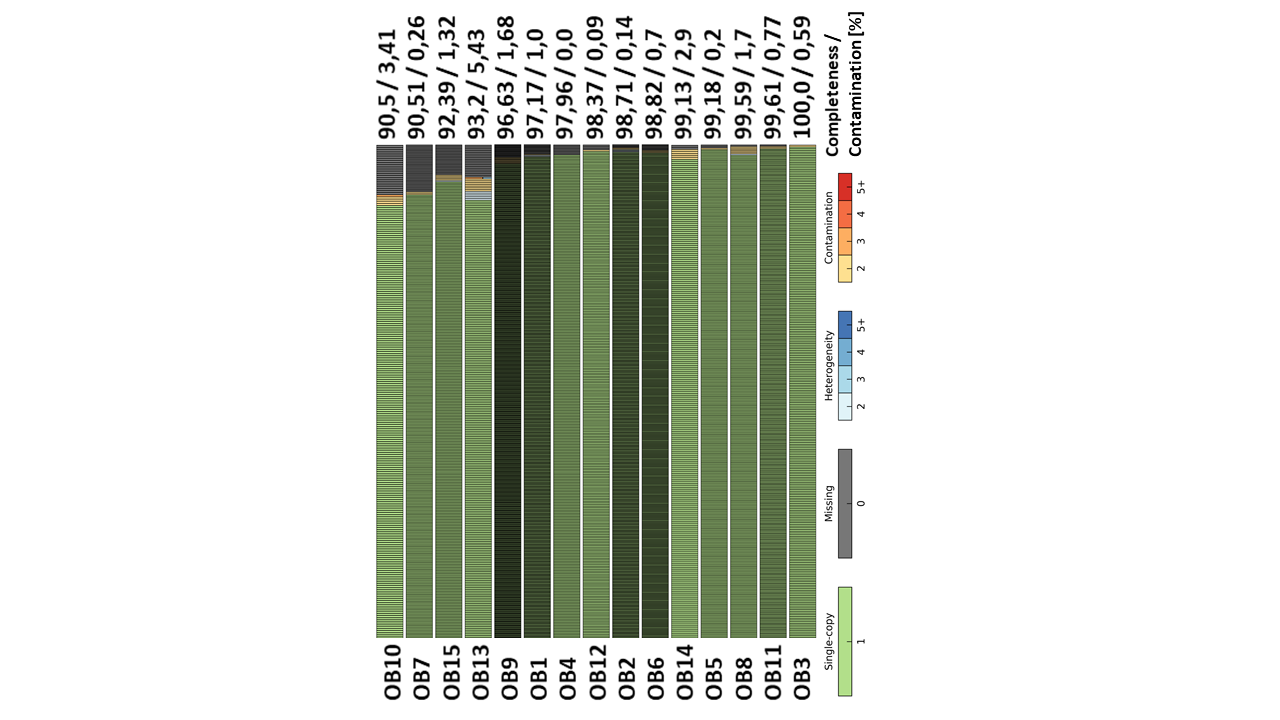


1. Quality control on CheckM with single copy marker genes of *Osedax* bone (OB) reconstructed MAGs.
